# Association study and a systematic meta-analysis of the VNTR polymorphism in the 3′-UTR of dopamine transporter gene and attention-deficit hyperactivity disorder

**DOI:** 10.1007/s00702-019-01998-x

**Published:** 2019-03-28

**Authors:** Edna Grünblatt, Anna Maria Werling, Alexander Roth, Marcel Romanos, Susanne Walitza

**Affiliations:** 1 Department of Child and Adolescent Psychiatry and Psychotherapy, University Hospital of Psychiatry Zurich, University of Zurich, Zurich, Switzerland; 2 Neuroscience Center Zurich, University of Zurich and ETH Zurich, Zurich, Switzerland; 3 Zurich Center for Integrative Human Physiology, University of Zurich, Zurich, Switzerland; 4 Department of Child and Adolescent Psychiatry, Psychosomatics and Psychotherapy, Center of Mental Health, University Hospital of Wuerzburg, Würzburg, Germany; 5 Translational Molecular Psychiatry, Department of Child and Adolescent Psychiatry and Psychotherapy, Centre for Child and Adolescent Psychiatry Research, University Hospital of Psychiatry Zurich, Wagistrasse 12, 8952 Schlieren, Switzerland

**Keywords:** Attention-deficit hyperactivity disorder, ADHD, Dopamine transporter, DAT1, *SLC6A3*, Meta-analysis

## Abstract

Attention-deficit hyperactivity disorder (ADHD) has been postulated to associate with dopaminergic dysfunction, including the dopamine transporter (DAT1). Several meta-analyses showed small but significant association between the 10-repeat allele in the *DAT1* gene in 3′-untranslated region variant number tandem repeat polymorphism and child and adolescent ADHD, whereas in adult ADHD the 9-repeat allele was suggested to confer as risk allele. Interestingly, recent evidence indicated that the long-allele variants (10 repeats and longer) might confer to lower expression of the transporter in comparison to the short-allele. Therefore, we assessed here the association in samples consisting of families with child and adolescent ADHD as well as a case–control sample, using either the 10- versus 9-repeat or the long- versus short-allele approach. Following, we conducted a systematic review and meta-analysis, including family and case–control studies, using the two aforementioned approaches as well as stratifying to age and ethnicity. The first approach (10-repeat) resulted in nominal significant association in child and adolescent ADHD (OR 1.1050 *p* = 0.0128), that became significant stratifying to European population (OR 1.1301 *p* = 0.0085). The second approach (long-allele) resulted in significant association with the whole ADHD population (OR 1.1046 *p* = 0.0048), followed by significant association for child and adolescent ADHD (OR 1.1602 *p* = 0.0006) and in Caucasian and in European child and adolescent ADHD (OR 1.1310 *p* = 0.0114; OR 1.1661 *p* = 0.0061; respectively). We were not able to confirm the association reported in adults using both approaches. In conclusion, we found further indication for a possible *DAT1* gene involvement; however, further studies should be conducted with stringent phenotyping to reduce heterogeneity, a limitation observed in most included studies.

**Electronic supplementary material:**

The online version of this article (10.1007/s00702-019-01998-x) contains supplementary material, which is available to authorized users.

## Introduction

Attention-deficit hyperactivity disorder (ADHD), characterized by persistent symptoms of inattention, hyperactivity and impulsivity, is one of the most common psychiatric and behavioural disorders in children and adolescents, with more than 5% of the paediatric population affected worldwide, and ADHD often persists into adulthood with a prevalence of 2.5–4.9% in adults (Thomas et al. 2015; Polanczyk et al. 2014). ADHD has been shown to have a high genetic component with around 80% heritability (Faraone and Larsson 2018). A recent genome-wide association study of over 20,000 ADHD patients has identified 12 independent loci to be genome-wide significantly associated with ADHD (Demontis et al. 2019). Nevertheless, genetic variations such as variable number tandem repeats (VNTRs) cannot be captured on such arrays, and therefore, conventional gene association studies may provide further information. One of these VNTRs (rs28363170), located on the dopamine transporter gene (*SLC6A3* / *DAT1*) in the 3′ untranslated region (UTR), with the 10- and 9-repeat alleles that are most common (Doucette-Stamm et al. 1995), were found to be associated with ADHD. In particular, the 10-repeat allele was described in several meta-analyses to associate with child and adolescent ADHD, though with a rather low effect size and in most cases with high heterogeneity between studies due to clinical phenotyping, age and ethnicity (Gizer et al. 2009; Yang et al. 2007). Nevertheless, further studies kept looking into the association between *DAT1* gene and ADHD, also due to significant linkage findings with the chromosomal location 5p13 containing the gene and ADHD (Friedel et al. 2007). Since it is known that there are further variants (3–13-repeats) on the *DAT1* 3′-UTR VNTR, expressing in various ethnicities in different frequencies (Mazei-Robinson and Blakely 2006), some studies looked also into associations with the 9- or the 11-repeat alleles. However, no association has been detected in child and adolescent ADHD both in European and Asian ethnicity (Li et al. 2006), whereas in adult ADHD a trend for association was found for 9-repeat allele carriers following a recent meta-analysis (Bonvicini et al. 2016).

The dopamine transporter is a key player in the dopaminergic system, regulating the synaptic dopamine homeostasis and its signalling. Since psychostimulants, such as amphetamine and methylphenidate, provide an effective treatment for ADHD (e.g., Faraone and Buitelaar 2010), are known to have high affinity to the transporter and inhibiting the transporter (Markowitz and Patrick 2008; Han and Gu 2006), the dopamine transporter has become one of the risk candidates for ADHD research. Dopamine controls numerous functions including attention, mood, cognition, reward and movement (Iversen and Iversen 2007). And altered dopamine homeostasis and particularly dopamine transporter is not exclusive for ADHD, but has been implicated in several disorders, including paediatric Bipolar Disorder (rs40184) (Mick et al. 2008), Major Depressive Disorder (9-repeat) (Lopez-Leon et al. 2008), Posttraumatic Stress Disorder (9-repeat) (Li et al. 2016), Tourette Syndrome (9-repeat associated with increased tics) (Tarnok et al. 2007), Obsessive–Compulsive Disorder (9-repeat) (Taylor 2013), Alzheimer’s disease (9-repeat) (Feher et al. 2014) and Alcoholism (9-repeat associated with alcohol withdrawal seizure and delirium tremens) (Du et al. 2011), while no association was found in Schizophrenia (Gamma et al. 2005)(see also meta-analysis in http://www.szgene.org) and Parkinson’s disease (Geissler et al. 2017) [for detailed review on dopamine transporter and various CNS disorders see (McHugh and Buckley 2015)]. However most studies demonstrated either conflicting findings or having only a single finding lacking replications or having no significant findings. In a meta-analysis of a collection of naturalistic studies ADHD children without 10/10-repeat genotypes responded better to methylphenidate; however, this effect was not found in clinically monitored studies (Soleimani et al. 2018). On the other hand, Parkinson’s disease carriers of the 9-repeat allele required lower levopoda doses, as well as were at risk of hallucination/psychosis following treatment (Politi et al. 2018). Similarly, in methamphetamine substance use, 9-repeat was shown to be a strong risk factor for a worse prognosis of methamphetamine psychosis (Ujike et al. 2003).

Investigation regarding the functional consequence of the aforementioned variants has shown some mixed results. In vitro, using various cell culture models and reporter gene designs, showed either the 9- or the 10-repeat to increase *DAT1* gene expression (Fuke et al. 2001; VanNess et al. 2005; Mill et al. 2005; Greenwood and Kelsoe 2003; Inoue-Murayama et al. 2002; Hill et al. 2010; Miller and Madras 2002). Ex vivo, gene expression in post-mortem brain as well as in periphery also showed some inconsistent results [3 monkey substantia nigra (SN) (Miller and Madras 2002], 20 post-mortem cerebellum and temporal lobe and 18 volunteers lymphocytes (Mill et al. 2002), 7 post-mortem midbrain (Brookes et al. 2007), post-mortem of 30 Alzheimer’s disease SN and polar region of the frontal lobe (Pinsonneault et al. 2011), post-mortem of the ventral midbrain from 18 controls and 18 cocaine users (Zhou et al. 2014). To our knowledge, no data are available linking between central nervous system and blood *DAT1* expression and *DAT1* genotypes. However, Wiers et al. (2018) reported correlations in a small post-mortem study investigating *DAT1* mRNA expression in SN of 3 adult ADHD and 13 controls and DAT protein expression in the Caudate. Indeed, they found a significant positive correlation between mRNA and protein expression in the two brain regions (Wiers et al. 2018). Furthermore, DNA methylation of the *DAT1* cluster A in blood was significantly correlated with the DNA methylation of the *DAT1* cluster A in SN (Wiers et al. 2018). In a recent meta-analysis of a collection of positron emission tomography (PET) a highly significant evidence for the 9-repeat allele was shown to be associated with increased dopamine-transporter activity in human adults, that was significant in healthy adults and only marginally significant in adult ADHD patients (Faraone et al. 2014). In the single-photon emission computed tomography (SPECT) analysis, although containing small sample size, similar results were obtained for healthy adults, while for affected adults (ADHD, Parkinson’s disease, Schizophrenia, and alcoholism) the opposite results were observed (Faraone et al. 2014). Interestingly, in adult age span (20–75 years of age) 10-repeat homozygotes showed reduced striatal activity during working memory task that reduced with age, that led to the hypothesis of earlier manifestation of cognitive impairment in 10-repeat homozygotes (Sambataro et al. 2015). Yet, most studies focused on the functional effects of the most frequent repeats, the 9- and 10-repeats, while few studies looked whether the other variants have any functional effect. In a recent study, the binding of HESR1 and two transcription factors, known to bind at the VNTR-site of the *DAT1* gene, was found to be inhibited depending on the length of the VNTR variant, with 11-repeats having low *DAT1* expression compared to the short variants up to 6-repeats (Kanno and Ishiura 2011). Moreover, the non-coding RNA, miR-491 was found to inhibit *DAT1* expression in a dose-dependent manner, in which higher repeat number (11-repeats) inhibited the expression compared to low number of repeats (up to 1-repeat) (Jia et al. 2016).

Therefore, in the current case–control and family study, we assessed whether the association between *DAT* 3′-UTR VNTR 10-repeat carriers or long-allele (10-repeats and higher) carriers conveys a risk for ADHD. In addition, we conducted a systematic review of the literature followed by an updated meta-analysis, including all current available associations with *DAT1* 3′-UTR VNTR for case–control and family studies in child and adolescent as well as adult ADHD in various ethnicities. To address the possible confounding effects by age of patients, and their ethnicity, a stratified meta-analysis for each variable was conducted.

## Materials and methods

### Study samples

Two hundred and two Caucasian nuclear families (146 families with both parents and 56 families with 1 parent) were recruited and the index patients were investigated in the in- and outpatient units and the day clinics of the Department of Child and Adolescent Psychiatry and Psychotherapy, University Hospital of Psychiatry Zurich. Families were included if at least the index patient fulfilled the diagnostic criteria for ADHD (F90.0 or F90.1) according to ICD-10 (World Health Organization 2016; Dilling et al. 1996). Accordingly, this resulted in total 738 individuals [202 index with ADHD (males = 153, females = 49); aged 12.62 ± 3.05; IQ 102.7 ± 13.2; 119 parents and sibs with ADHD (males = 41, females = 78) and 417 control parents and sibs (males = 194, females = 223)]. The ADHD diagnoses of the parents and siblings were reported by the parents. The clinical diagnostic assessment of the index patient was done by a child and adolescent psychiatrist or psychologist under supervision of a senior psychiatrist in the clinic. The index patient was required to be ≥ 6 years old and to have an IQ over 75 as assessed with either the Wechsler Intelligence Scale for Children (WISC) (Wechsler 1991; Tewes et al. 1999), the Kaufman Assessment Battery for Children (K-ABC) (Kaufman and Kaufman 1983; Melchers and Preuss 1994), the Culture Fair Test (CFT-20-R) (Weiss 2006), Snijders-Oomen Nonverbal Intelligence Test (SON-R) (Tellegen et al. 2003) or Intelligence and Development Scales (IDS) (Grob et al. 2009). Exclusion criteria were (a) no Caucasian origin, (b) potentially confounding and severe psychiatric diagnoses such as psychosis, any pervasive developmental disorder, primary mood or anxiety disorder and Tourette’s syndrome, (c) neurological disorders such as epilepsy, (d) a history of any acquired brain damage or evidence of the fetal alcohol syndrome, (e) premature deliveries (delivery before 37th gestational week) and/or (f) maternal reports of severe prenatal, perinatal or postnatal complications.

In the case–control setting, the 220 index patients (males = 164, females = 56; aged 12.7 ± 2.98; IQ 103.2 ± 13.6) were compared to genetically independent 158 Caucasian healthy controls (males = 89, females = 69, aged 11.52 ± 3.091; IQ 111.5 ± 13.3) who were recruited at the Departments of Child and Adolescent Psychiatry of the Universities of Wuerzburg and Zurich. The cases in the case–control study consist of some index from the family study with additional new cases without family members recruited. Informed written consent was obtained in all cases from the participants and their parents. The study was approved by the ethical commissions of both of the involved universities in accordance with the latest version of the Declaration of Helsinki, including an ethical permission granted by the Ethic Committees from Wuerzburg, and the Cantonal Ethic Committee of Zurich (Ref. Nr. KFO 140/03 and KEK-ZH-Nr. 2016-00101).

### Genotyping

DNA was isolated either from whole blood collected in ethylenediaminetetraacetic acid (EDTA) tubes using QIAamp DNA Blood Maxi Kit (Qiagen), or from saliva collected in the Oragene DNA collection kit (DNA Genotek, Canada) and isolated as per manufacturer’s protocol. DNA concentrations, A260/A280, and A260/A230 ratios were measured using a spectrophotometer (NanoVue Plus, GE). The study population was genotyped for the DAT1 3′-UTR VNTR polymorphism, using the primers F: 5′-TGTGGTGTAGGGAACGGCCTGAG-3′ and R: 5′-CTTCCTGGAGGTCACGGCTCAAGG-3′. A total volume of 25 µl containing 1 µl DNA sample (50 ng/µl) were mixed with 12.5 µl Promega GoTaq^®^ Green Master Mix (Promega), 9.5 µl water and 1 µl of both primers (forward and reverse 10 µM). The reaction was performed in 0.2 ml PCR 8-tube strips, sealed with individually attached caps (Bio-Rad) in a C1000™ CFX96™ Thermal cycler (Bio-Rad). DNA amplification was achieved under the following conditions: 2 min at 95 °C followed by 30 cycles at 95 °C for 45 s, 67.5 °C for 45 s, 72 °C for 1 min, and a final extension of 5 min at 72 °C. 15 µl of PCR product was loaded into a 3% agarose gel (SeaKem^®^ LE Agarose mixed with 1x TAE buffer) stained with 5 µl HDGreen™ Plus DNA Stain (Intas) per 100 g agarose and run for 90 min at 120 V. A 100 bp BenchTop DNA ladder (Promega) was used to assess the size of the PCR product and set the genotypes according to the expected bands of 316 bp for 6-repeat allele, 396 bp for 8-repeat allele, 436 bp for 9-repeat allele, 476 bp for 10-repeat allele and 516 bp for 11-repeat allele. PCR were run for sample DNA samples in duplicates to ensure reproducibility. In case of ambiguity in the duplicates, genotyping was repeated in a separate run to resolve the discrepancy. No-template controls (NTC) were included in every run to exclude impurities.

### Statistical analysis

All association studies were run on the PLINK v1.7 [URL: http://pngu.mgh.harvard.edu/purcell/plink/ (Purcell et al. 2007)]. Each study group (case–control study) was tested for Hardy–Weinberg equilibrium (HWE) that confirmed no deviations from HWE for all samples. For the case–control association study Fisher’s Exact Test was conducted and significance was set at *p* < 0.025 as Bonferroni correction was conducted (analysis of the *DAT1* in two forms: long-allele vs. short-allele and 10-repeat vs. 9-repeat). For the family association study, Mendel errors test (none were found) followed by the transmission disequilibrium test (TDT) was conducted as well as a parent-of-origin analysis.

### Search strategy and study selection

A systematic literature search was conducted to include studies that examined associations of *DAT1* 3′-UTR VNTR polymorphisms with ADHD. We searched PubMed and Web of Science databases for articles published until December 31, 2018. Literature was searched using the keywords: (DAT* OR SLC6A3 OR “dopamine transporter”) AND (polymorphism* OR VNTR OR “tandem repeat” OR “untranslated region”) AND (ADHD OR “attention hyperactivity”). As a further search manner, we searched the reference sections of the most recently published studies identified in literature search described above, as well as any recently published systematic review articles and meta-analysis, to identify studies that might have been missed. The Preferred Reporting Items for Systematic Reviews and Meta-Analyses (PRISMA) flow diagram (Liberati et al. 2009) was used to report the search flow for this meta-analysis. Studies included in our meta-analysis had to fulfil the following criteria: (1) detailed description of the sample size, ancestry of participants, and diagnostic criteria for ADHD, (2) case–control or family studies examining the association between *DAT1* 3′-UTR VNTR polymorphism and ADHD, and (3) containing data on allele/genotype frequencies in case and control groups and/or odds ratio (OR) and 95% confidence interval (CI) for the OR, (4) samples not duplicative of other studies. In addition to performing a meta-analysis of all studies pooled together, we conducted a subgroup analysis by dividing the studies into those including patients with Caucasian or Asian ancestry to examine the effects of ethnic heterogeneity as well as for adult versus child and adolescent ADHD. If articles only reported allele/genotype frequencies, OR and 95% CI was calculated from allele frequencies using an online OR calculator (https://www.genecalculators.net/associatorrr-cc.html). Corresponding authors and co-authors were contacted in case of missing data in the selected publications, and were asked to provide the missing information. In case of no respond, articles were excluded (*n* = 4).

### Data synthesis and statistical analysis

The quality of all included studies was assessed based on traditional epidemiological considerations for genetic studies as previously described in Liu et al. (2015; Supplementary Table S1). The meta-analysis was conducted using the MIX 2.0-Professional software for meta-analysis in Excel, version 2.0.1.6 (http://www.meta-analysis-made-easy.com/) (Bax et al. 2006). The OR of each study was converted to the natural logarithm of OR [Ln (OR)], and 95% CI to standard error (SE) using MIX 2.0 software. The Ln (OR), SE, and sample size (*N*) were used in the software to perform heterogeneity statistics, heterogeneity funnel plots, and synthesis forest plots using inverse variance weighting. The heterogeneity between studies was assessed by Cochran’s Chi square-based *Q* statistic and the inconsistency index (*I*^2^), with *p* < 0.05 being considered statistically significant. If there was significant (*p* < 0.05) heterogeneity between studies, we used the random-effects model, otherwise we used the fixed-effects model. The random-effects model considers both between-study and within-study variation, whereas the fixed-effects model considers only within-study variation (Borenstein et al. 2010). The heterogeneity *I*^2^ are presented in Supplementary Tables S2 and S3. Potential publication bias was assessed using Begg’s test (Begg and Mazumdar 1994) and Egger’s regression test (Egger et al. 1997), with *p* < 0.05 considered statistically significant (Supplementary Tables S2 and S3). In case of *p* < 0.05 for publication bias, the trim and fill correction was conducted to explore the corrected OR and 95% CI (Duval and Tweedie 2000).

## Results

### DAT1 3′-UTR VNTR association with child and adolescent ADHD in the Zurich samples

In the case–control study a nominal significant association between *DAT1* 3′-UTR VNTR long-allele and ADHD was observed (OR 0.697, 95% CI 0.501–0.972, *p* = 0.03). Furthermore, a significant association was found when assessing according to 10-repeat allele versus 9-repeat allele carriers (OR 0.676, 95% CI 0.484–0.945, *p* = 0.024). Test statistic was based on 211 cases and 155 controls, and genotypes are presented in Table 1a.

**Table 1:** Genotype distribution of the *DAT1* 3′-UTR VNTR in both Zurich samples

(a) Case–control study sample						
	*DAT1* 3′-UTR VNTR genotypes					
	9/9	10/9	11/9	10/10	10/11	11/11
ADHD	22	88	2	101	6	1
Control	9	54	1	90	2	0

(b) Family study sample						
	*DAT1* 3′-UTR VNTR genotypes					
	9/9	10/9	11/9	10/10	10/11	11/11
ADHD	19	82	1	94	5	1
Mother	28	80	2	84	2	1
Father	8	53	1	87	2	1
Sibs	13	84	0	93	2	0

Family-based association analyses of the *DAT1* 3′-UTR VNTR long-allele as well as the 10-repeat versus 9-repeat allele yielded no significant association in the Zurich sample (OR 1.015, 95% CI 0.726–1.418, *p* = 0.932; OR 1.048, 95% CI 0.742–1.480, *p* = 0.792; respectively). Data from 46 heterozygous parents were included to assess the TDT, and genotypes are presented in Table 1b.

### Meta-analysis for *DAT1* 3′-UTR VNTR association with child, adolescent, and adult ADHD in different ethnical groups

The literature search for studies reporting on the association of *DAT1* 3′-UTR VNTR with ADHD identified 899 non-duplicated articles (Supplementary Figure S1). Out of 861 articles, 767 articles were excluded at the title/abstract level. Altogether, 100 articles were read fully, and 34 articles were excluded because various reasons: e.g., missing data, overlapping samples in other publications or not fulfilling the inclusion criteria (Supplementary Table S4). Finally, 61 publications were included in the meta-analysis (Supplementary Table S5 including studies characteristics, demographics and quality assessment scores), including the current study results (Cook et al. 1995; Waldman et al. 1998; Jiang et al. 1999; Lunetta et al. 2000; Swanson et al. 2000; Curran et al. 2001; Todd et al. 2001; Maher et al. 2002; Smith et al. 2003; Carrasco et al. 2004; Kustanovich et al. 2004; Galili-Weisstub et al. 2005; Bakker et al. 2005; Bobb et al. 2005; Feng et al. 2005; Kim et al. 2005, 2006; Langley et al. 2005; Simsek et al. 2005; Hawi et al. 2005, 2010; Brookes et al. 2006; Cheuk et al. 2006; Hebebrand et al. 2006; Lim et al. 2006; Asherson et al. 2007; Brüggemann et al. 2007; Qian et al. 2007; Genro et al. 2008; Johansson et al. 2008; Wang et al. 2008; Banoei et al. 2008; Kopeckova et al. 2008; Franke et al. 2008, 2010; Niederhofer et al. 2008; Kereszturi et al. 2008; Gizer et al. 2009; Wohl et al. 2008; Martinez-Levy et al. 2009, 2013; Dresler et al. 2010; Aparecida da Silva et al. 2011; Bidwell et al. 2011; Das et al. 2011; El-Tarras et al. 2012; Hoogman et al. 2013; de Azeredo et al. 2014; Shang and Gau 2014; Hasler et al. 2015; Sery et al. 2015; Fonseca et al. 2015; Agudelo et al. 2015; Gomez-Sanchez et al. 2016; Onnink et al. 2016; Ortega-Rojas et al. 2017; Stanley et al. 2017; Wiguna et al. 2017; Hong et al. 2018; Morgan et al. 2018). The summary of OR, SE, type of study (e.g., family TDT, case–control CC etc.), number of participants, age of cases (child and adolescent or adult ADHD), ethnicity/country, and the ID given for all studies included are presented in Supplementary Tables S6 and S7 (10-repeats vs. 9-repeats, and Long-allele vs. Short-allele, respectively). The meta-analyses summary of all *DAT1* 3′-UTR VNTR analysis variations (long-allele vs. short-allele; 10-repeat vs. 9-repeat) with the entire publications, stratified to age of cases (child and adolescent or adult ADHD) and ethnical grouping is presented in Supplementary Tables S2 and S3.

We found no significant association between 10-repeat allele carriers and the entire ADHD population, as well as after stratifying to adult ADHD (Supplementary Tables S2). However, stratification to child and adolescent ADHD resulted in a nominal significant association (OR 1.1050 95% CI 1.0203–1.1968, *p* = 0.0128), nevertheless the analysis was accompanied with high heterogeneity that was not due to publications bias. Following ethnical stratification, children and adolescent ADHD originating from Europe demonstrated a significant association with 10-repeat allele as risk allele (OR 1.1301 95% CI 1.0316–1.2379, *p* = 0.0085), nevertheless, also here a significant heterogeneity was found. For further detailed stratifications results see Supplementary Table S2.

**Fig. 1:**
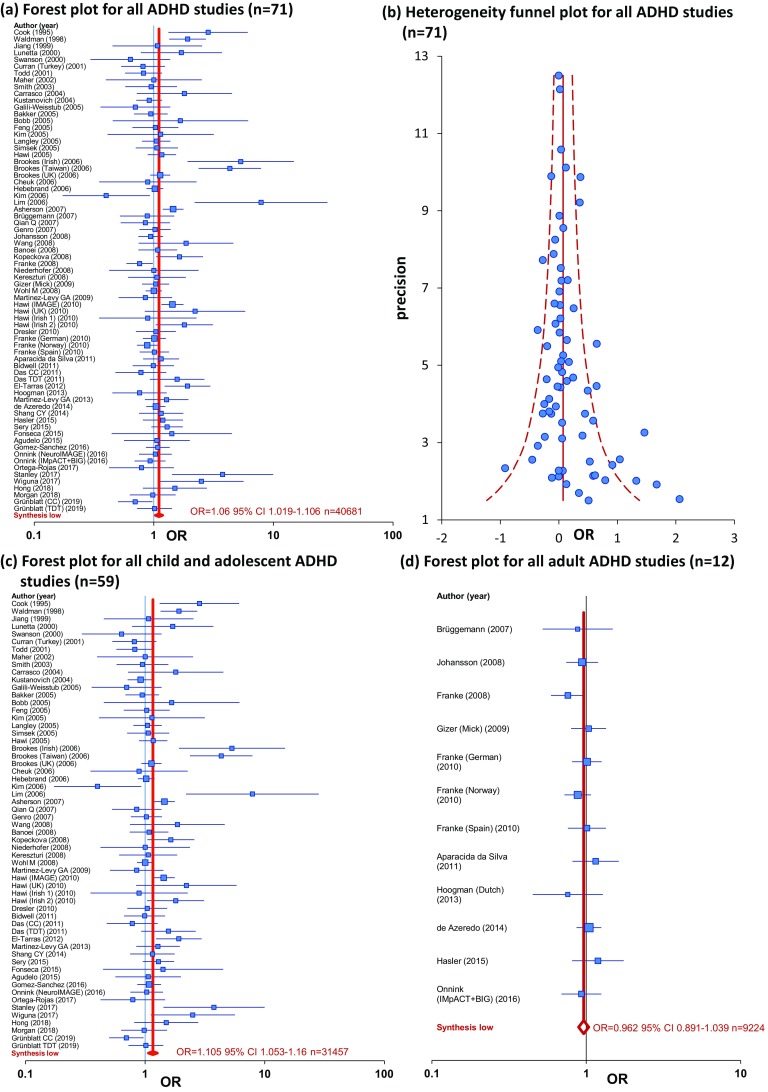
Meta-analysis of all cohorts and published association analyses (*n* = 71) of the *DAT1* 3′-UTR VNTR Long-allele with attention-deficit hyperactivity disorder (ADHD) (**a**). Heterogeneity funnel plot assessing any evidence of publication bias for whole ADHD studies *I*^2^ = 54.167% (95% CI 40.03–64.97%) *p* = 0 (**b**). Forest plot in child and adolescent ADHD studies (*n* = 59) (**c**). Forest plot in adult ADHD studies (*n* = 12) (**d**). Black whiskers in the forest plot represent 95% confidence intervals (CI) for odds ratio; the weight (inverse variance) of the study is reflected in symbol (box) size. Sample demographics, individual statistics, heterogeneity, literature bias statistics, quality assessments and scores, and model used is summarized in Supplementary Tables S3, S5–S6. The order of the samples is as presented in the Supplementary Tables and in Supplementary Figures S2–S4, in a descending manner

**Fig. 2:**
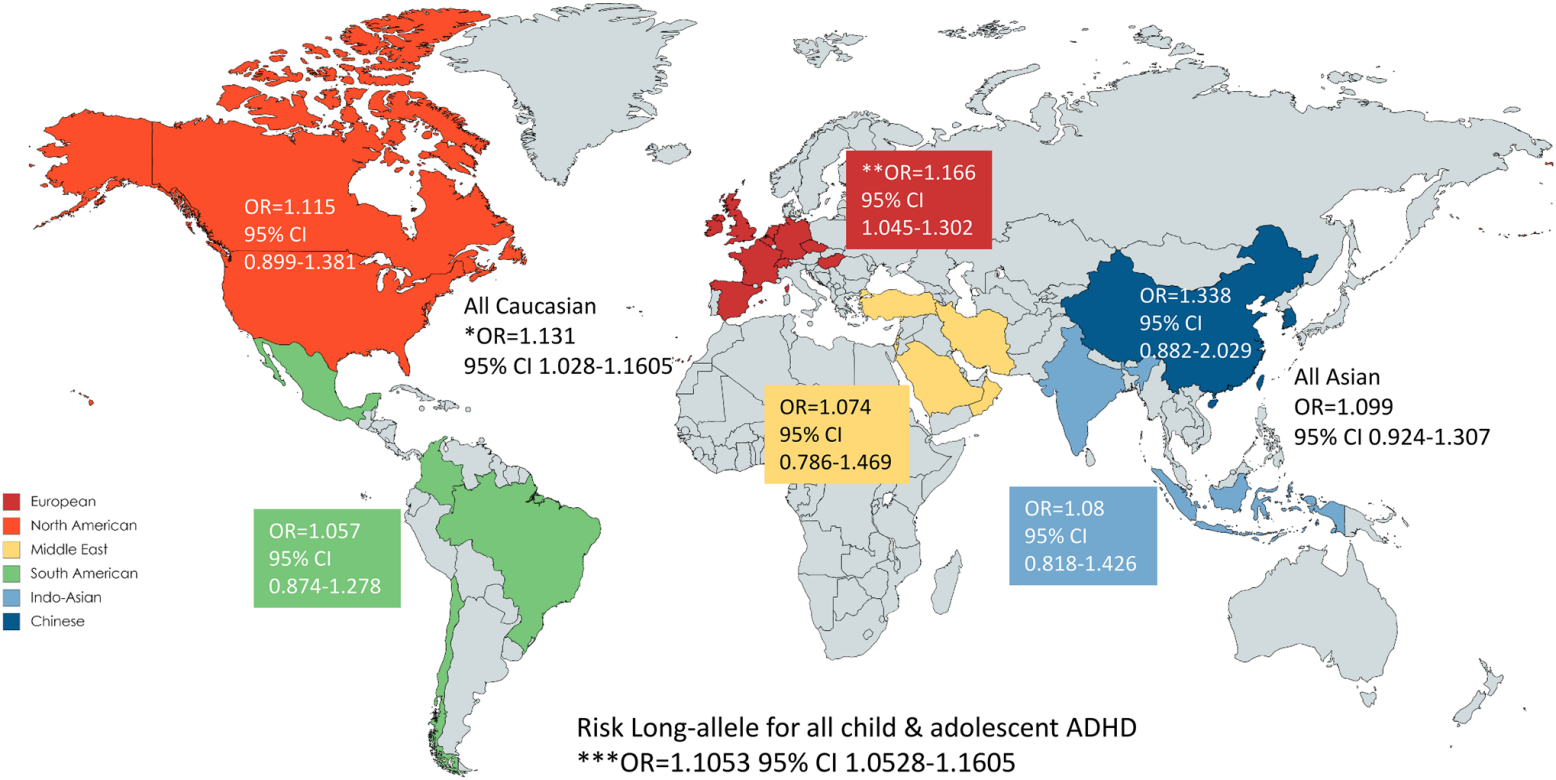
World map overview of the countries that were included in the child and adolescent ADHD meta-analysis (*DAT1* 3′-UTR VNTR Long-repeat allele as risk allele) according to the various stratifications. The odds ratio (OR) and 95% CI are displayed for each ethnicity category. *p* value < 0.05*; < 0.01**; < 0.001***

*DAT1* 3′-UTR VNTR long-allele was significantly associated with ADHD assessed as the whole ADHD population (OR 1.1046 95% CI 1.0309–1.1837, *p* = 0.0048). Similarly to the 10-repeat analysis, significant heterogeneity was observed with publication bias. Following trim and fill correction the association was still significant with Long-allele as risk allele (OR 1.0614 95% CI 1.0186–1.1060). Following stratification with age, only child and adolescent ADHD kept the significant association (OR 1.1602 95% CI 1.0657–1.2631, *p* = 0.0006; Trim and Fill OR 1.1053 95% CI 1.0525–1.1605), however still with high heterogeneity. In Caucasian child and adolescent ADHD, and in European child and adolescent ADHD, a significant association with the Long-allele was found (OR 1.1310 95% CI 1.0282–1.2441, *p* = 0.0114; OR 1.1661 95% CI 1.0448–1.3015, *p* = 0.0061; respectively). In the whole Asian population a nominal significant association was observed, however following trim and fill correction significance was lost (OR 1.0991 95% CI 0.9240–1.3074). The forest and heterogeneity funnel plot for the whole ADHD sample is presented in Fig. 1a, b, and the forest plot results following stratification to age (child and adolescent-, adult-ADHD) is presented in Fig. 1c, d. Summary of the meta-analysis results for the ethnicity stratification is demonstrated in the world map in Fig. 2.

## Discussion

The current meta-analysis, could confirm previous findings showing weak association between the 10-repeat allele of the *DAT1* 3′-UTR VNTR gene and child and adolescent ADHD, that reached significance only in the European population; however, this was accompanied by high heterogeneity that was in some cases due to literature bias but in other cases due to heterogeneity in clinical phenotyping, age or ethnicity (Li et al. 2006; Gizer et al. 2009). On the other hand, we were not able to confirm a significant association between the 9-repeat allele and adult ADHD as previously reported (Bonvicini et al. 2016), and only a nominal significant association was observed for the European adult ADHD with 9-repeat as risk allele (see Supplementary Table S2).

Interestingly, assessing the functional approach, in which the long-allele was suggested to result in decreased dopamine transporter expression (Faraone et al. 2014; Kanno and Ishiura 2011; Jia et al. 2016), seem to show significant association with ADHD. Indeed, the meta-analysis including the overall ADHD studies, as well as the child and adolescent ADHD, the Caucasian child and adolescent ADHD, the European child and adolescent ADHD, and nominally the Asian child and adolescent ADHD resulted in significant association with Long-allele as risk allele. In the other ethnical groups the associations did not reach significance, however all seem to show some tendency toward the long-allele as risk allele, however further studies should be conducted to confirm this hypothesis.

The current study included the largest sample size available (total *n* = 40,681 consisting of 14,821 cases) for a powerful meta-analysis of the *DAT1* 3′-UTR VNTR gene variants, and used when needed the random-effects model that incorporate heterogeneity among trials (Borenstein et al. 2010). Indeed, following quality assessment of all included studies according to traditional epidemiological considerations (Liu et al. 2015) indicated that some studies did not reach high quality scores due to sample size, diagnostic criteria, recruitment strategies and quality control of the genetic analysis. This was reflected in significant heterogeneity, that in some cases, was also confirmed with significant publication bias assessed with Begg’s test (Begg and Mazumdar 1994) and Egger’s regression test (Egger et al. 1997). In these few cases we corrected the results using trim and fill correction (Duval and Tweedie 2000), that kept their significance even after correction.

To summarize the current study, we could show further evidence of the *DAT1* 3′-UTR VNTR variants to play a role in ADHD, in particularly in child and adolescents with the Long-allele as risk allele. As previously hypothesized, this could indeed be due to the possible functional effect of the variants length in controlling the expression of the *DAT1* gene. However, as still high study heterogeneity was observed, with some studies not reaching high quality, further analysis is necessary to establish a robust conclusion.

## Electronic supplementary material

Below is the link to the electronic supplementary material.

Supplementary material 1 (PDF 2756 KB)

Supplementary material 2 (PDF 438 KB)

Supplementary material 3 (PDF 439 KB)
