# Supplementary material for: Association study and a systematic meta-analysis of the VNTR polymorphism in the 3′-UTR of dopamine transporter gene and attention-deficit hyperactivity disorder
Source: J Neural Transm (Vienna). 2019 Mar 28;126(4):517–29. doi: 10.1007/s00702-019-01998-x (PMC6456487; doi:10.1007/s00702-019-01998-x)
Supplement: Supplementary file 1 — Supplementary material 1 (PDF 2756 KB) [file 702_2019_1998_MOESM1_ESM.pdf]

Supplementary material

Association study and a systematic meta-analysis of the VNTR polymorphism in the 3'-UTR of dopamine transporter gene and attention-deficit hyperactivity disorder

Edna Grünblatt[1,2,3] ✉, Anna Maria Werling[1], Alexander Roth[1], Marcel Romanos[4], Susanne Walitza[1,2,3]

[1] Department of Child and Adolescent Psychiatry and Psychotherapy, University Hospital of Psychiatry Zurich, University of Zurich, Zurich, Switzerland

[2] Neuroscience Center Zurich, University of Zurich and ETH Zurich, Zurich, Switzerland

[3] Zurich Center for Integrative Human Physiology, University of Zurich, Zurich, Switzerland

[4] Center of Mental Health, Department of Child and Adolescent Psychiatry, Psychosomatics and Psychotherapy, University Hospital of Wuerzburg, Würzburg, Germany

Supplementary table S1 Quality assessment scores of each study taken into the meta-analysis (according to the Score sheet from Liu et al. (Liu et al. 2015)

A)

Criteria	Cook et al. 1995	Waldman et al. 1998	Jiang et al. 1999	Lunetta et al. 2000	Swanson et al. 2000	Curran et al. 2001 (Turkey)	Todd et al. 2001	Maher et al. 2002	Smith et al. 2003	Carrasco et al. 2004	Kustanovich et al. 2004	Galili-Weisstub et al. 2005	Bakker et al. 2005	Bobb et al. 2005 TDT	Feng et al, 2005	Kim et al. 2005	Langley et al. 2005 CC
**Source of cases**																	
Selected from population or general registry (Score 3)			3					3									
Selected from hospital (Score 2)	2	2			2	2	2		2	2	2	2	2	2	2	2	2
Selected from pathology archives, but without description (Score 1)																	
Not described (Score 0)				0													
**Source of controls (when family study the score is automatic 3)**																	
Population-based (Score 3)	3	3	3	3	3	3	3	3		3	3	3	3	3	3	3	3
Hospital-based (Score 1.5)																	
Not described (Score 0)									0								
**Diagnostic criteria of ADHD**																	
Acknowledged operational diagnostic criteria, such as DSM-IV, ICD-10 (Score 3)	3	3	3	3	3	3	3	3		3	3	3	3	3	3	3	3
Patient medical record (Score 1.5)									1.5								
Not description (Score 0)																	
**Case-control match (when family study the score is automatic 3)**																	
Matched by age and gender (Score 3)	3	3	3	3	3	3	3	3	3	3	3	3	3	3	3	3	
Matched only by age or by gender (Score 1.5)																	
Not matched or not described (Score 0)																	0
**Specimens used for determining genotypes**																	
Peripheral blood or buccal swabs (Score 3)	3	3	3					3	3	3	3	3	3	3	3	3	3
Not description (Score 0)				0	0	0	0										
**Quality control of genotyping**																	
Different genotyping assays confirmed the result (Score 3)	3																
Quality control by repeated assay (Score 1.5)		1.5											1.5				
Not description (Score 0)			0	0	0	0	0	0	0	0	0	0		0	0	0	0
**Hardy-Weinberg equilibrium in controls (when family study the score is automatic 3)**																	
Hardy-Weinberg equilibrium (Score 3)	3	3	3	3	3	3	3	3	3	3	3	3	3	3	3	3	3
Hardy-Weinberg disequilibrium (Score 0)																	
**Sample size**																	
≧300 (Score 3)		3				3	3				3		3	3	3	3	3
≧200 and ﹤300 (Score 2)			2									2					
≧100 and ﹤200 (Score 1)	1								1								
﹤100 (Score 0)				0				0		0							
**Total (Score 0-24)**	21	21.5	20	12	14	17	17	18	13.5	17	20	19	21.5	20	20	20	17

B) Continue

Criteria	Simsek et al. 2005	Hawi et al. 2005	Brookes et al. 2006 Taiwan	Brookes et al. 2006 UK	Brookes et al. 2006 IMAGE ST1	Cheuk et al. 2006 TDT	Hebebrand et al. 2006	Kim et al. 2006 CC	Lim et al. 2006	Asherson et al. 2007 IMAGE ST2	Brüggermann et al. 2007	Qian Q et al. 2007	Genro et al. 2007	Johansson et al. 2008	Wang et al. 2008	Banoei et al. 2008	Kopeckova et al. 2008
**Source of cases**																	
Selected from population or general registry (Score 3)														3			
Selected from hospital (Score 2)	2	2	2	2	2	2	2	2	2	2		2	2		2	2	
Selected from pathology archives, but without description (Score 1)																	
Not described (Score 0)											0						0
**Source of controls (when family study the score is automatic 3)**																	
Population-based (Score 3)	3	3	3	3	3	3	3		3	3		3	3	3	3		
Hospital-based (Score 1.5)								1.5									
Not described (Score 0)											0					0	0
**Diagnostic criteria of ADHD**																	
Acknowledged operational diagnostic criteria, such as DSM-IV, ICD-10 (Score 3)	3	3	3	3	3	3	3	3	3	3		3	3	3	3	3	3
Patient medical record (Score 1.5)																	
Not description (Score 0)											0						
**Case-control match (when family study the score is automatic 3)**																	
Matched by age and gender (Score 3)		3	3	3	3	3	3		3	3			3	3	3		
Matched only by age or by gender (Score 1.5)	1.5							2								1.5	1.5
Not matched or not described (Score 0)											0	0					
**Specimens used for determining genotypes**																	
Peripheral blood or buccal swabs (Score 3)	3				3	3		3	3			3	3	3	3	3	3
Not description (Score 0)		0	0	0			0			0	0						
**Quality control of genotyping**																	
Different genotyping assays confirmed the result (Score 3)																	
Quality control by repeated assay (Score 1.5)							1.5							1.5			
Not description (Score 0)	0	0	0	0	0	0		0	0	0	0	0	0		0	0	0
**Hardy-Weinberg equilibrium in controls (when family study the score is automatic 3)**																	
Hardy-Weinberg equilibrium (Score 3)	3	3	3	3	3	3	3	3	3	3			3	3	3	3	3
Hardy-Weinberg disequilibrium (Score 0)											0	0					
**Sample size**																	
≧300 (Score 3)		3	3	3	3		3			3		3	3	3			
≧200 and < 300 (Score 2)	2										2					2	2
≧100 and < 200 (Score 1)						1		1							1		
< 100 (Score 0)									0								
**Total (Score 0-24)**	17.5	17	17	17	20	18	18.5	15.5	17	17	2	14	20	22.5	18	14.5	12.5

C) Continue

Criteria	Franke et al. 2008	Niederhofer et al. 2008	Keresztnuri et al. 2008	Gizer (Mick) et al 2009	Wohl M et al. 2008	Martinez-Levy GA et al. 2009	Hawi et al. 2010 (IMAGE)	Hawi et al. 2010 (English 3)	Hawi et al. 2010 (Irish 2)	Hawi et al. 2010 (Irish 1)	Dresler et al. 2010	Franke et al. 2010 (German)	Franke et al. 2010 (Norway)	Franke et al. 2010 (Spanish)	Aparacida da Silva et al. 2011	Bidwell et al. 2011	Das et al. 2011 (CC)	Das et al. 2011 (TDT)
**Source of cases**																		
Selected from population or general registry (Score 3)													3			3		
Selected from hospital (Score 2)	2		2	2	2	3	2	2	2	2	2	2		2	2		2	2
Selected from pathology archives, but without description (Score 1)																		
Not described (Score 0)		0																
**Source of controls (when family study the score is automatic 3)**																		
Population-based (Score 3)	3	3	3	3	3	3	3	3	3	3	3	3	3	3	3	3		3
Hospital-based (Score 1.5)																		
Not described (Score 0)																	0	
**Diagnostic criteria of ADHD**																		
Acknowledged operational diagnostic criteria, such as DSM-IV, ICD-10 (Score 3)	3	3	3	3	3	3	3	3	3	3	3	3	3	3	3	3	3	3
Patient medical record (Score 1.5)																		
Not description (Score 0)																		
**Case-control match (when family study the score is automatic 3)**																		
Matched by age and gender (Score 3)		3	3	3	3	3	3	3	3	3	3				3	3		3
Matched only by age or by gender (Score 1.5)	1.5																	
Not matched or not described (Score 0)												0	0	0			0	
**Specimens used for determining genotypes**																		
Peripheral blood or buccal swabs (Score 3)		3	3			3					3	3	3		3	3	3	3
Not description (Score 0)	0			0	0		0	0	0	0				0				
**Quality control of genotyping**																		
Different genotyping assays confirmed the result (Score 3)												3	3	3				
Quality control by repeated assay (Score 1.5)	1.5					1.5												
Not description (Score 0)		0	0	0	0		0	0	0	0	0				0	0	0	0
**Hardy-Weinberg equilibrium in controls (when family study the score is automatic 3)**																		
Hardy-Weinberg equilibrium (Score 3)	3		3	3	3	3	3	3	3	3	3	3	3	3	3		3	3
Hardy-Weinberg disequilibrium (Score 0)		0														0		
**Sample size**																		
≧300 (Score 3)	3		3	3	3		3			3		3	3	3	3	3		3
≧200 and ﹤300 (Score 2)						2		2			2						2	
≧100 and ﹤200 (Score 1)		1							1									
﹤100 (Score 0)																		
**Total (Score 0-24)**	17	13	20	17	17	21.5	17	16	15	17	19	20	21	17	20	18	13	20

D) Continue

Criteria	El-Tarras et al. 2012	Hoogman et al. 2013 (IMpACT)	Martinez-Levy GA et al. 2013	de Azeredo et al. 2014	Shang CY et al. 2014	Hasler et al. 2015	Sery et al. 2015	Fonseca et al. 2015 TDT	Agudelo et al. 2015	Gomez-Sanchez et al. 2016	Onnink et al. 2016 (NeuroIMAGE)	Onnink et al. 2016 (IMpACT+BIG)	Ortega-Rojas et al. 2017	Stanley et al. 2017	Wiguna et al. 2017	Hong et al. 2018	Morgan et al. 2018	Grünblatt et al CC	Grünblatt et al TDT
**Source of cases**																			
Selected from population or general registry (Score 3)			3						3		3	3							
Selected from hospital (Score 2)	2	2		2	2	2	2			2			2	2	2	2	2	2	2
Selected from pathology archives, but without description (Score 1)																			
Not described (Score 0)								0											
**Source of controls (when family study the score is automatic 3)**																			
Population-based (Score 3)		3	3		3	3	3		3	3	3	3	3		3	3	3	3	3
Hospital-based (Score 1.5)	1.5			1.5										1.5					
Not described (Score 0)								0											
**Diagnostic criteria of ADHD**																			
Acknowledged operational diagnostic criteria, such as DSM-IV, ICD-10 (Score 3)	3	3	3	3	3	3	3	3	3	3	3	3	3	3	3	3	3	3	3
Patient medical record (Score 1.5)																			
Not description (Score 0)																			
**Case-control match (when family study the score is automatic 3)**																			
Matched by age and gender (Score 3)		3			3		3	3		3	3	3	3		3	3		3	3
Matched only by age or by gender (Score 1.5)				1.5		1.5								1.5			1.5		
Not matched or not described (Score 0)	0		0						0										
**Specimens used for determining genotypes**																			
Peripheral blood or buccal swabs (Score 3)	3	3	3	3		3	3	3	3	3	3	3	3	3	3	3	3	3	3
Not description (Score 0)					0														
**Quality control of genotyping**																			
Different genotyping assays confirmed the result (Score 3)						3								3					
Quality control by repeated assay (Score 1.5)									1.5									1.5	1.5
Not description (Score 0)	0	0	0	0	0		0	0		0	0	0	0		0	0	0		
**Hardy-Weinberg equilibrium in controls (when family study the score is automatic 3)**																			
Hardy-Weinberg equilibrium (Score 3)			3	3	3			3	3	3	3	3			3		3	3	3
Hardy-Weinberg disequilibrium (Score 0)	0	0				0	0						0	0		0			
**Sample size**																			
≧300 (Score 3)			3	3	3	3	3			3	3	3				3		3	3
≧200 and ﹤300 (Score 2)	2							2					2				2		
≧100 and ﹤200 (Score 1)		1							1										
﹤100 (Score 0)														0	0				
**Total (Score 0-24)**	11.5	15	18	17	17	18.5	17	14	17.5	20	21	21	16	14	17	17	17.5	21.5	21.5

Supplementary table S4 Excluded studies in the *DAT1* 3'-UTR VNTR meta-analyses and reasons for the exclusion

Serial No.	References	Reasons
1	(Gill et al. 1997)	Excluded as the cohort was enlarged in Daly et al. 1999
2	(Daly et al. 1999)	Excluded as the cohort was enlarged in Hawi et al. 2003
3	(Palmer et al. 1999)	Excluded as the cohort was enlarged in Kustanovich et al. 2004
4	(Holmes et al. 2000)	Excluded as the cohort was enlarged in Langley et al. 2005
5	(Barr et al. 2001)	Excluded as the cohort was enlarged in Feng et al. 2005
6	(Curran et al. 2001)- **only the UK population**	Excluded as the UK cohort was enlarged in Brookes et al. 2006
7	(Roman et al. 2001)	Excluded as the cohort was enlarged in Genro et al. 2007
8	(Rowe et al. 2001)	Excluded as the sample has no diagnose for ADHD (retrospective)
9	(Kirley et al. 2002)	Excluded as the cohort already reported in Daly et al. 1999
10	(DiMaio et al. 2003)	Excluded as only a review
11	(Chen et al. 2003)	Excluded as the cohort was enlarged in Brookes et al. 2006
12	(Hawi et al. 2003)	Excluded as the cohort was enlarged in Hawi et al. 2005
13	(Qian et al. 2004)	Excluded as no info was available from authors and the cohort was enlarged in Qian et al. 2007
14	(Wang et al. 2004)	Excluded as the cohort was already published in Wang et al. 2008
15	(Purper-Ouakil et al. 2005)	Excluded as is a meta-analysis without any original unpublished data
16	(Bobb et al. 2005)- **only case-control**	Excluded as the Cases are already in TDT
17	(Cornish et al. 2005)	Excluded as related only to epidemiological samples with no DSM or ICD diagnosis
18	(Langley et al. 2005)- **only the FBAT sample**	Excluded as cases already included in the case-control and in FBAT no information to OR was available
19	(Todd et al. 2005)	Excluded as the cohort was already published in Kustanovich 2004, Palmer 1999 Todd 2001
20	(Cheuk et al. 2006)- **only case-control**	Excluded as the Cases are already in TDT
21	(Kim et al. 2006)- **only the TDT sample**	Excluded as did not get any info from authors while the Cases are already in TDT
22	(Carrasco et al. 2006)	Excluded as the cohort was the same as in Carrasco et al. 2004
23	(Li et al. 2006)	Excluded as is a meta-analysis without any original unpublished data
24	(Yang et al. 2007)	Excluded as is a meta-analysis without any original unpublished data
25	(Das and Mukhopadhyay 2007)	Excluded as the cohort was enlarged in Das et al. 2011
26	(Nyman et al. 2007)	Excluded as No response from any of the authors
27	(Kereszturi et al. 2008)- **only case-control**	Excluded as the Cases are already in TDT, as well as controls were unmatched
28	(Langley et al. 2009)	Excluded as the sample already described in Langley et al. 2005
29	(Franke et al. 2010)- **only Brüggeman and Dutch samples**	Excluded as already described in Brüggemann 2007, as well as in Franke et al. 2008
30	(Costa et al. 2011)	Excluded as related only to dopamine binding
31	(Brown et al. 2011)	Excluded due to failing information not possible to extract as first and last author are not available
32	(Shang et al. 2011)	Excluded as cohort was enlarged in Shang et al. 2014
33	(Spencer et al. 2013)	Fischman passed away, while the others claimed not to have the data (Spencer could not be reached)
34	(Faraone et al. 2014)	Excluded as describes meta-analysis with PET and SPECT

35	(Kambeitz et al. 2014)	Excluded as describes meta-analysis of *DAT1* 3'-UTR VNTR and methylphenidate response
36	(van Rooij et al. 2015)	Excluded as most likely is the same sample as in Onnink et al 2016
37	(Gatt et al. 2015)	Excluded as only a review
38	(Ettinger et al. 2016)	Excluded as described meta-analysis between *DAT1* 3'-UTR VNTR and cognition without diagnosis information
39	(Bonvicini et al. 2016)	Excluded as describes only meta-analysis in adults ADHD without original unpublished data
40	(Soleimani et al. 2018)	Excluded as describes meta-analysis of *DAT1* 3'-UTR VNTR and methylphenidate response
41	(Adriani et al. 2018)	Excluded as related only to methylation data

Supplementary table S5 Characteristics of included studies in the *DAT1* 3'-UTR VNTR meta-analyses

Serial No.	Reference	Country [ethnicity]	Study design	ADHD [Child-adolescent/ Adult]	Diagnostic criteria	Case/control [family]	Quality score**	HWE
1	(Cook et al. 1995)	USA (83% Caucasian)	HHRR	Children	DSM-III-R	[49]	21	n.a.
2	(Waldman et al. 1998)	USA (68% Caucasian)	TDT	Children	DSM-IV	[117]	21.5	yes
3	(Jiang et al. 1999)	Chinese	HRR	Children	DSM-III-R	[74]	20	yes
4	(Lunetta et al. 2000)	USA	TDT	Children	DSM-IV	[35]	12	n.a.
5	(Swanson et al. 2000)	USA	HRR	Children	DSM-IV	[80]	14	n.a.
6	(Curran et al. 2001)	Turkey	TDT	Children	DSM-IV	[111]	17	n.a.
7	(Todd et al. 2001)	USA	TDT	Children	DSM-IV	[523]	17	n.a.
8	(Maher et al. 2002)	USA Caucasian (males)	TDT	Children	DSM-III-R	[33]	18	n.a.
9	(Smith et al. 2003)	USA Caucasian	CC	Children	Semi DSM-IV	105/68	13.5	yes
10	(Carrasco et al. 2004)	Chile	HHRR	Children	DSM-IV	[26]	17	n.a.
11	(Kustanovich et al. 2004)	USA (79% Caucasian)	TDT	Children	DSM-IV	[293]	20	n.a.
12	(Galili-Weisstub et al. 2005)	Jewish	HRR	Children	DSM-IV	[68]	19	n.a.
13	(Bakker et al. 2005)	Dutch	TDT	Children	DSM-IV	[236]	21.5	yes
14	(Bobb et al. 2005)	USA (75% Caucasian)	TDT	Children	DSM-IV	[163]	20	yes
15	(Feng et al. 2005)	Canada	TDT	Children	DSM-IV	[178]	20	n.a.
16	(Kim et al. 2005)	Korea	TDT	Children	DSM-IV	[107]	20	yes
17	(Langley et al. 2005)	UK (White British)	CC	Children	ICD-10, DSM-IV & DSM-III-R	259/278	17	yes
18	(Simsek et al. 2005)	Oman	CC	Children	DSM-IV	92/110	17.5	yes
19	(Hawi et al. 2005)	Ireland	TDT	Children	DSM-IV	[179]	17	n.a.
20	(Brookes et al. 2006)	Taiwan	TDT	Children	DSM-IV	[216]	17	n.a.
		UK	TDT	Children	DSM-IV	[180]	17	n.a.
		European (ST1 IMAGE; 8 countries)	TDT	Children	DSM-IV	[664]	20	yes
21	(Cheuk et al. 2006)	Hong Kong	TDT	Children	DSM-IV	[64]	18	n.a.
22	(Hebebrand et al. 2006)	German	TDT	Children	ICD10 & DSM-IV	[102]	18.5	n.a.
23	(Kim et al. 2006)	Korea	CC	Children (Control- Mix of children and adults)	DSM-IV	85/100	15.5	yes
24	(Lim et al. 2006)	Korea	HHRR	Children	DSM-IV	[33]	17	n.a.

25	(Asherson et al. 2007)	European (ST2 IMAGE; 8 countries)	TDT	Children	DSM-IV	[998]	17	n.a.
26	(Brüggemann et al. 2007)	German	CC	Adults	n.a.	122/177	2	n.a.
27	(Qian et al. 2007)	Chinese (Han)	CC	Children	DSM-IV	332/216	14	yes
28	(Genro et al. 2008)	Brazil (92% Caucasian)	TDT	Children	DSM-IV	[241]	20	yes
29	(Johansson et al. 2008)	Norwegian	CC	Adults	ICD-10 DSM-IV	358/340	22.5	yes
30	(Wang et al. 2008)	Chinese (Han)	HHRR	Children	DSM-IV	[54]	18	yes
31	(Banoei et al. 2008)	Iran	CC	Children	DSM-IV	100/130	14.5	yes
32	(Kopeckova et al. 2008)	Czech	CC	Children	DSM-IV	100/100	12.5	yes
33	(Franke et al. 2008)	Dutch	CC	Adults	DSM-IV	213/507	17	yes
34	(Niederhofer et al. 2008)	European (German/Austria)	HRR	Children	DSM-IV	[49]	13	n.a.
35	(Kereszturi et al. 2008)	Hungarian	TDT	Children	DSM-IV	[168]	20	yes
36	(Gizer et al. 2009)	USA (97% Caucasian)	TDT	Children & Adults	DSM-III-R / DSM-IV	[266]	17	n.a.
37	(Wohl et al. 2008)	French (86% Caucasian)	TDT	Children	DSM-IV TR	[146]	17	n.a.
38	(Martinez-Levy et al. 2009)	Mexico	CC	Children	ICD-10, DSM-IV-R	105/113	21.5	yes
39	(Hawi et al. 2010)	IMAGE (European)	TDT	Children	DSM-IV	[1033]	17	n.a.
		English	TDT	Children	DSM-IV	[107]	16	n.a.
		Irish (2)	TDT	Children	DSM-IV	[52]	15	n.a.
		Irish (1)	TDT	Children	DSM-IV	[178]	17	n.a.
40	(Dresler et al. 2010)	German	CC	Adults	DSM-IV	161/109	19	n.a.
41	(Franke et al. 2010)	German	CC	Adults	DSM-IV	421/405	20	yes
		Norwegian	CC	Adults	DSM-IV	450/548	21	yes
		Spanish (Caucasian)	CC	Adults	DSM-IV	264/195	17	yes
42	(Aparecida da Silva et al. 2011)	Brazil	CC	Adults	DSM-IV	102/479	20	yes
43	(Bidwell et al. 2011)	USA	HHRR	Children	DSM-IV	[202]	18	n.a.

44	(Das et al. 2011)	India	CC	Children (Control- Mix)	DSM-IV	125/93	13	yes
		India	TDT	Children	DSM-IV	[122]	20	yes
45	(El-Tarras et al. 2012)	Saudi	CC	Children	DSM-IV	120/160	11.5	n.a.
46	(Hoogman et al. 2013)	Dutch	CC	Adults	DSM-IV	87/77	15	n.a.
47	(Martinez-Levy et al. 2013)	Mexico	CC	Children	ICD-10, DSM-IV	215/283	18	yes
48	(de Azeredo et al. 2014)	Brazil (European)	CC	Adults	DSM-IV	476/587	17	yes
49	(Shang and Gau 2014)	Taiwan	TDT	Children	DSM-IV	[382]	17	yes
50	(Hasler et al. 2015)	European (Swiss)	CC	Adults	DSM-IV TR	75/391	18.5	n.a.
51	(Sery et al. 2015)	Czech	CC	Children	ICD-10, DSM-IV	218/253	17	n.a.
52	(Fonseca et al. 2015)	Columbia	TDT	Children	DSM-IV	[86]	14	yes
53	(Agudelo et al. 2015)	Columbia	CC	Children	DSM-IV	70/53	17.5	yes
54	(Gomez-Sanchez et al. 2016)	Spanish	CC	Children	DSM-IV TR	290/340	20	yes
55	(Onnink et al. 2016)	Dutch (NeuroIMAGE)	CC	Children	DSM-IV	301/186	21	yes
		Dutsch (IMpACT+BIG)	CC	Adults	DSM-IV	118/111+1718	21	yes
56	(Ortega-Rojas et al. 2017)	Columbia	TDT	Children	DSM-IV TR	[97]	16	n.a.
57	(Stanley et al. 2017)	India	CC	Children	DSM-5	44/44	14	n.a.
58	(Wiguna et al. 2017)	Indonesian	CC	Children	DSM-IV TR	47/48	17	yes
59	(Hong et al. 2018)	Korea	CC	Children	DSM-IV	150/322	17	n.a.
60	(Morgan et al. 2018)	USA (53% Caucasian)	CC	Children	DSM-IV, DISC	113/99	17.5	yes
61	Current study	Caucasian	CC	Children	ICD-10, DSM-IV & DSM-5	220/156	21.5	yes
		Caucasia	TDT	Children	ICD-10, DSM-IV & DSM-5	[202]	21.5	yes

Abbreviations: CC, case-control study; TDT, transmission disequilibrium test; FBAT, Family Based Association Test; HRR family-based study using haplotype relative risk, HHRR, haplotype-based haplotype relative risk; n.a., not available; ADHD, attention-deficit hyperactivity disorder; DSM-IIIR/IV, diagnostic and statistical manual of mental disorders, fourth Edition/ third revised Edition; ICD-10, international classification of disease; HWE, hardy-Weinberg equilibrium; DISC, Diagnostic Interview Schedule for Children-IV.

** See table Quality assessment score (Supplementary Table S3); according to the Score sheet from Liu et al. ((Liu et al. 2015) as following:

Low quality scores	<16
High quality scores	>16
Middle quality score	=16

Supplementary table S6 Summary of all studies included in the meta analysis for the association between *DAT1* 3'-UTR VNTR 10-repeat allele and 9-repeat allele.

id	author	year	type	N	N Cases	OR	CI-	CI+	log(or)	se	Ancestry	Child/Adult
1	Cook	1995	HHRR	147	49	2.842	1.324	6.099	1.045	0.390	USA (83% Caucasian)	Child
2	Waldman	1998	TDT	392	117	1.915	1.346	2.725	0.650	0.180	USA (68% Caucasian)	Child
3	Jiang	1999	HRR	222	74	1.070	0.450	2.530	0.068	0.440	Chinese	Child
4	Lunetta	2000	TDT	60	33	1.700	0.778	3.713	0.531	0.399	USA	Child
5	Swanson	2000	HRR	240	80	0.636	0.295	1.371	-0.452	0.391	USA	Child
6	Curran (Turkey)	2001	TDT	333	111	0.813	0.533	1.240	-0.208	0.216	Turkey	Child
7	Todd	2001	TDT	2092	219	0.821	0.575	1.173	-0.197	0.182	USA	Child
8	Maher	2002	TDT	99	33	1.000	0.397	2.519	0.000	0.471	Caucasian (male)	Child
9	Smith	2003	CC	173	105	0.950	0.580	1.570	-0.051	0.254	Caucasian (USA)	Child
10	Carrasco	2004	HHRR	51	26	1.800	0.719	4.505	0.588	0.468	Chile	Child
11	Kustanovich	2004	TDT	1120	535	0.915	0.714	1.174	-0.088	0.127	USA (79% Caucasian)	Child
12	Galili-Weisstub	2005	HRR	204	68	1.031	0.634	1.677	0.031	0.248	Jewish	Child
13	Bakker	2005	TDT	901	434	0.944	0.684	1.304	-0.057	0.165	Dutch	Child
14	Bobb	2005	TDT	355	163	1.667	0.452	6.144	0.511	0.666	USA (75% Caucasian)	Child
15	Feng	2005	TDT	541	226	1.000	0.728	1.374	0.000	0.162	Canada	Child
16	Kim	2005	TDT	321	107	1.063	0.537	2.103	0.061	0.348	Korean	Child
17	Langley	2005	CC	537	259	1.047	0.800	1.380	0.046	0.139	UK (white British)	Child
18	Simsek	2005	CC	179	84	1.130	0.730	1.750	0.122	0.223	Oman	Child
19	Hawi	2005	TDT	537	179	1.167	0.889	1.531	0.154	0.139	Irish	Child
20	Brookes	2006	TDT	475	216	3.111	1.468	6.593	1.135	0.383	Taiwan	Child
21	Brookes	2006	TDT	476	180	2.031	1.330	3.102	0.709	0.216	UK	Child
22	Brookes	2006	TDT	2748	776	1.130	0.931	1.372	0.122	0.099	European (ST1 IMAGE; 8 countries)	Child
23	Cheuk	2006	TDT	192	64	0.890	0.350	2.280	-0.117	0.478	Chinese (Hong Kong)	Child
24	Hebebrand	2006	TDT	425	229	1.021	0.868	1.199	0.020	0.082	German	Child
25	Kim	2006	CC	185	85	0.317	0.142	0.709	-1.149	0.411	Korean	Child
26	Lim	2006	HHRR	99	33	7.880	2.200	28.290	2.064	0.652	Korean	Child

27	Asherson	2007	TDT	3352	1159	1.205	1.045	1.389	0.186	0.073	European (ST2 IMAGE; 8 countries)	Child
28	Brüggemann	2007	CC	296	122	0.880	0.520	1.480	-0.128	0.267	German	Adults
29	Qian Q	2007	CC	476	284	0.660	0.360	1.210	-0.416	0.309	Chinese (Han)	Child
30	Genro	2007	TDT	689	241	1.024	0.759	1.380	0.023	0.152	Brazil (92% Caucasian)	Child
31	Johansson	2008	CC	698	358	0.940	0.740	1.190	-0.062	0.121	Norway	Adults
32	Wang	2008	HHRR	111	51	1.200	0.370	3.910	0.182	0.601	Chinese (Han)	Child
33	Banoei	2008	CC	230	100	1.078	0.743	1.565	0.075	0.190	Iran	Child
34	Kopeckova	2008	CC	200	100	1.640	1.045	2.576	0.495	0.230	Czech	Child
35	Franke	2008	CC	720	213	0.760	0.590	0.980	-0.274	0.129	Dutch	Adults
36	Niederhofer	2008	HRR	134	49	1.000	0.420	2.350	0.000	0.439	German/Austria	Child
37	Kereszturi	2008	TDT	440	168	1.325	0.625	2.811	0.281	0.384	Hungarian	child
38	Gizer (Mick)	2009	TDT	1006	474	1.036	0.798	1.345	0.035	0.133	USA (97% Caucasian)	Child/Adult
39	Wohl M	2008	TDT	398	146	1.000	1.000	1.000	0.000	0.080	French (86% Caucasian)	Child
40	Martinez-Levy GA	2009	CC	218	105	0.850	0.510	1.430	-0.163	0.263	Mexico	Child
41	Hawi	2010	TDT	3099	1033	1.490	1.200	1.840	0.399	0.109	IMAGE (European)	Child
42	Hawi	2010	TDT	277	107	2.210	0.840	5.820	0.793	0.494	English	Child
43	Hawi	2010	TDT	156	52	0.780	0.300	2.060	-0.248	0.491	Irish 2	Child
44	Hawi	2010	TDT	534	178	1.630	0.930	2.850	0.489	0.286	Irish 1	Child
45	Dresler	2010	CC	270	161	1.040	0.710	1.530	0.039	0.196	German (Würzburg)	Child
46	Franke (IMpACT)	2010	CC	826	421	1.010	0.810	1.260	0.010	0.113	German (Würzburg)	Adults
47	Franke (IMpACT)	2010	CC	998	450	0.880	0.720	1.070	-0.128	0.101	Norway	Adults
48	Franke (IMpACT)	2010	CC	459	264	1.010	0.760	1.340	0.010	0.145	Spanish (Caucasian)	Adults
49	Aparacida da Silva	2011	CC	555	99	1.090	0.770	1.530	0.086	0.175	Brazil	Adults
50	Bidwell	2011	HHRR	606	202	0.990	0.670	1.480	-0.010	0.202	USA	Child
51	Das	2011	CC	211	120	0.880	0.430	1.800	-0.128	0.365	Indian	Child
52	Das	2011	TDT	354	122	0.400	0.180	0.890	-0.916	0.408	Indian	Child
53	El-Tarras	2012	CC	135	30	0.670	0.360	1.270	-0.400	0.322	Saudi	Child
54	Hoogman (IMpACT)	2013	CC	160	83	0.600	0.360	0.990	-0.511	0.258	Dutch	Adults
55	Martinez-Levy GA	2013	CC	622	215	1.280	0.840	1.940	0.247	0.214	Mexico	Child

56	de Azeredo	2014	CC	1063	476	1.040	0.870	1.260	0.039	0.094	Brazil (European)	Adults
57	Shang CY	2014	TDT	1298	439	1.150	0.750	1.760	0.140	0.218	Taiwan	Child
58	Hasler	2015	CC	372	70	0.900	0.590	1.370	-0.105	0.215	European (Swiss)	Adults
59	Sery	2015	CC	471	218	1.290	0.950	1.740	0.255	0.154	Czech	Child
60	Fonseca	2015	TDT	258	86	1.412	0.223	2.252	0.345	0.590	Columbia (Spanish)	Child
61	Agudelo	2015	CC	123	70	1.060	0.560	1.980	0.058	0.322	Columbia (Spanish)	Child
62	Gomez-Sanchez	2016	CC	630	290	0.970	0.770	1.210	-0.030	0.115	Spanish (Caucasian)	Child
63	Onnink (NeuroIMAGE)	2016	CC	471	294	1.050	0.760	1.440	0.049	0.163	Dutch	Child
64	Onnink (IMpACT+BIG)	2016	CC	1894	115	0.830	0.610	1.120	-0.186	0.155	Dutch	Adults
65	Ortega-Rojas	2017	TDT	291	97	0.787	0.424	1.471	-0.239	0.317	Columbia (Spanish)	Child
66	Stanley	2017	CC	85	42	4.930	1.580	15.340	1.595	0.580	Indian	Child
67	Wiguna	2017	CC	92	47	2.400	1.050	5.460	0.875	0.421	Indonesian	Child
68	Hong	2018	CC	450	146	1.530	0.650	3.620	0.425	0.438	Korean	Child
69	Morgan	2018	CC	212	113	0.980	0.630	1.520	-0.020	0.225	53% Caucasian	Child
70	Grünblatt	2019	CC	366	211	0.676	0.484	0.945	-0.391	0.171	Caucasian (Swiss)	Child
71	Grünblatt	2019	TDT	719	314	1.048	0.742	1.480	0.047	0.176	Caucasian (Swiss)	Child

Supplementary table S7 Summary of all studies included in the meta analysis for the association between *DAT1* 3'-UTR VNTR Long-repeat allele and Short-repeat allele.

id	author	year	type	N	N Cases	OR	CI-	CI+	log(or)	se	Ancestry	Child/Adult
1	Cook	1995	HHRR	147	49	2.842	1.324	6.099	1.045	0.390	USA (83% Caucasian)	Child
2	Waldman	1998	TDT	392	117	1.915	1.346	2.725	0.650	0.180	USA (68% Caucasian)	Child
3	Jiang	1999	HRR	222	74	1.070	0.450	2.530	0.068	0.440	Chinese	Child
4	Lunetta	2000	TDT	60	33	1.700	0.778	3.713	0.531	0.399	USA	Child
5	Swanson	2000	HRR	240	80	0.636	0.295	1.371	-0.452	0.391	USA	Child
6	Curran (Turkey)	2001	TDT	333	111	0.813	0.533	1.240	-0.208	0.216	Turkey	Child
7	Todd	2001	TDT	2092	219	0.821	0.575	1.173	-0.197	0.182	USA	Child
8	Maher	2002	TDT	99	33	1.000	0.397	2.519	0.000	0.471	Caucasian (male)	Child
9	Smith	2003	CC	173	105	0.950	0.580	1.570	-0.051	0.254	Caucasian (USA)	Child
10	Carrasco	2004	HHRR	51	26	1.800	0.719	4.505	0.588	0.468	Chile	Child
11	Kustanovich	2004	TDT	1120	535	0.915	0.714	1.174	-0.088	0.127	USA (79% Caucasian)	Child
12	Galili-Weisstub	2005	HRR	204	68	0.700	0.360	1.380	-0.357	0.343	Jewish	Child
13	Bakker	2005	TDT	901	434	0.944	0.684	1.304	-0.057	0.165	Dutch	Child
14	Bobb	2005	TDT	355	163	1.667	0.452	6.144	0.511	0.666	USA (75% Caucasian)	Child
15	Feng	2005	TDT	541	226	1.030	0.660	1.600	0.030	0.226	Canada	Child
16	Kim	2005	TDT	321	107	1.140	0.410	3.150	0.131	0.520	Korean	Child
17	Langley	2005	CC	537	259	1.047	0.800	1.380	0.046	0.139	UK (white British)	Child
18	Simsek	2005	CC	202	92	1.060	0.710	1.600	0.058	0.207	Oman	Child
19	Hawi	2005	TDT	537	179	1.167	0.889	1.531	0.154	0.139	Irish	Child
20	Brookes	2006	TDT	475	216	5.320	1.910	14.770	1.671	0.522	Taiwan	Child
21	Brookes	2006	TDT	476	180	4.320	2.370	7.890	1.463	0.307	UK	Child
22	Brookes	2006	TDT	2748	776	1.130	0.931	1.372	0.122	0.099	European (ST1 IMAGE; 8 countries)	Child
23	Cheuk	2006	TDT	192	64	0.890	0.350	2.280	-0.117	0.478	Chinese (Hong Kong)	Child
24	Hebebrand	2006	TDT	425	229	1.021	0.868	1.199	0.020	0.082	German	Child
25	Kim	2006	CC	185	85	0.400	0.170	0.910	-0.916	0.428	Korean	Child
26	Lim	2006	HHRR	99	33	7.880	2.200	28.290	2.064	0.652	Korean	Child

27	Asherson	2007	TDT	3352	1159	1.450	1.190	1.770	0.372	0.101	European (ST2 IMAGE; 8 countries)	Child
28	Brüggemann	2007	CC	296	122	0.880	0.520	1.480	-0.128	0.267	German	Adults
29	Qian Q	2007	CC	548	332	0.850	0.530	1.370	-0.163	0.242	Chinese (Han)	Child
30	Genro	2007	TDT	689	241	1.024	0.759	1.380	0.023	0.152	Brazil (92% Caucasian)	Child
31	Johansson	2008	CC	698	358	0.940	0.740	1.190	-0.062	0.121	Norway	Adults
32	Wang	2008	HHRR	162	54	1.860	0.750	4.640	0.621	0.465	Chinese (Han)	Child
33	Banoei	2008	CC	230	100	1.078	0.743	1.565	0.075	0.190	Iran	Child
34	Kopeckova	2008	CC	200	100	1.640	1.045	2.576	0.495	0.230	Czech	Child
35	Franke	2008	CC	720	213	0.760	0.590	0.980	-0.274	0.129	Dutch	Adults
36	Niederhofer	2008	HRR	134	49	1.000	0.420	2.350	0.000	0.439	German/Austria	Child
37	Keresztúri	2008	TDT	440	168	1.060	0.610	1.860	0.058	0.284	Hungarian	child
38	Gizer (Mick)	2009	TDT	1006	474	1.036	0.798	1.345	0.035	0.133	USA (97% Caucasian)	Child/Adult
39	Wohl M	2008	TDT	398	146	1.000	1.000	1.000	0.000	0.080	French (86% Caucasian)	Child
40	Martinez-Levy GA	2009	CC	218	105	0.850	0.510	1.430	-0.163	0.263	Mexico	Child
41	Hawi	2010	TDT	3099	1033	1.430	1.150	1.760	0.358	0.109	IMAGE (European)	Child
42	Hawi	2010	TDT	277	107	2.210	0.840	5.820	0.793	0.494	English	Child
43	Hawi	2010	TDT	156	52	0.890	0.350	2.280	-0.117	0.478	Irish 2	Child
44	Hawi	2010	TDT	534	178	1.800	1.040	3.120	0.588	0.280	Irish 1	Child
45	Dresler	2010	CC	270	161	1.040	0.710	1.530	0.039	0.196	German (Würzburg)	Child
46	Franke (IMpACT)	2010	CC	826	421	1.010	0.810	1.260	0.010	0.113	German (Würzburg)	Adults
47	Franke (IMpACT)	2010	CC	998	450	0.880	0.720	1.070	-0.128	0.101	Norway	Adults
48	Franke (IMpACT)	2010	CC	459	264	1.010	0.760	1.340	0.010	0.145	Spanish (Caucasian)	Adults
49	Aparacida da Silva	2011	CC	581	102	1.150	0.810	1.620	0.140	0.177	Brazil	Adults
50	Bidwell	2011	HHRR	606	202	0.990	0.670	1.480	-0.010	0.202	USA	Child
51	Das	2011	CC	218	125	0.780	0.480	1.280	-0.248	0.250	Indian	Child
52	Das	2011	TDT	354	122	1.565	0.928	2.641	0.448	0.267	Indian	Child
53	El-Tarras	2012	CC	280	120	1.910	1.230	2.960	0.647	0.224	Saudi	Child
54	Hoogman (IMpACT)	2013	CC	164	87	0.760	0.450	1.280	-0.274	0.267	Dutch	Adults
55	Martinez-Levy GA	2013	CC	622	215	1.280	0.840	1.940	0.247	0.214	Mexico	Child

56	de Azeredo	2014	CC	1063	476	1.040	0.870	1.260	0.039	0.094	Brazil (European)	Adults
57	Shang CY	2014	TDT	1298	439	1.150	0.750	1.760	0.140	0.218	Taiwan	Child
58	Hasler	2015	CC	466	75	1.190	0.810	1.750	0.174	0.197	European (Swiss)	Adults
59	Sery	2015	CC	471	218	1.290	0.950	1.740	0.255	0.154	Czech	Child
60	Fonseca	2015	TDT	258	86	1.412	0.223	2.252	0.345	0.590	Columbia (Spanish)	Child
61	Agudelo	2015	CC	123	70	1.060	0.560	1.980	0.058	0.322	Columbia (Spanish)	Child
62	Gomez-Sanchez	2016	CC	630	290	1.080	0.860	1.360	0.077	0.117	Spanish (Caucasian)	Child
63	Onnink (NeuroIMAGE)	2016	CC	487	301	1.030	0.750	1.410	0.030	0.161	Dutch	Child
64	Onnink (IMpACT+BIG)	2016	CC	1947	118	0.930	0.690	1.250	-0.073	0.152	Dutch	Adults
65	Ortega-Rojas	2017	TDT	291	97	0.787	0.424	1.471	-0.239	0.317	Columbia (Spanish)	Child
66	Stanley	2017	CC	88	44	3.760	1.420	9.950	1.324	0.497	Indian	Child
67	Wiguna	2017	CC	95	47	2.500	1.110	5.610	0.916	0.413	Indonesian	Child
68	Hong	2018	CC	471	150	1.500	0.810	2.780	0.405	0.315	Korean	Child
69	Morgan	2018	CC	212	113	0.980	0.630	1.520	-0.020	0.225	53% Caucasian	Child
70	Grünblatt	2019	CC	376	220	0.697	0.501	0.972	-0.360	0.169	Caucasian (Swiss)	Child
71	Grünblatt	2019	TDT	743	324	1.015	0.726	1.418	0.015	0.171	Caucasian (Swiss)	Child

PRISMA 2009 Flow Diagram

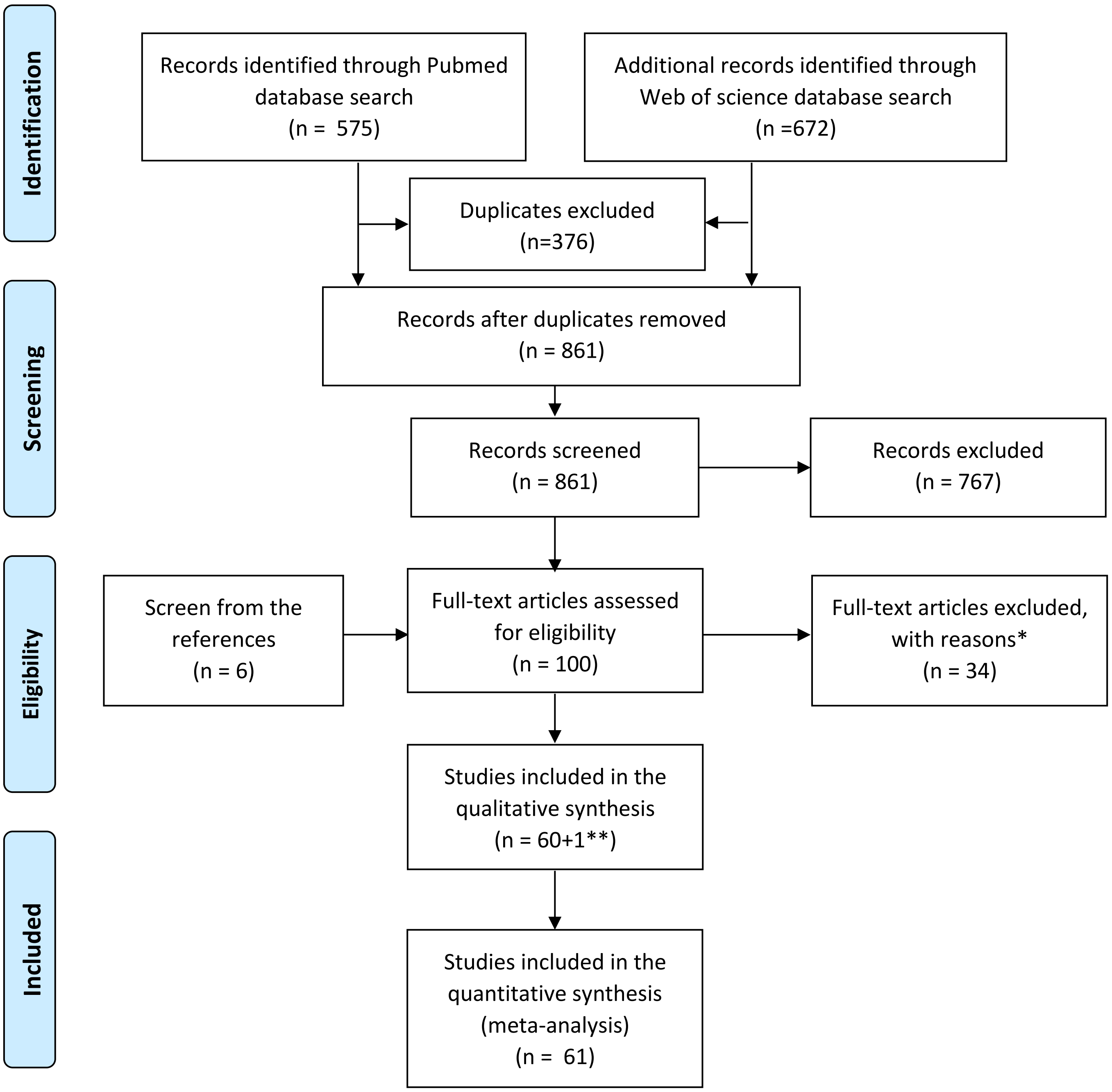

Supplementary Figure S1 PRISMA Flow diagram for literature search of *DAT1* 3'-UTR VNTR polymorphism and ADHD data published till December 31, 2018. Search terms were (DAT* OR SLC6A3 OR "dopamine transporter") AND (polymorphism* OR VNTR OR "tandem repeat" OR "untranslated region") AND (ADHD OR "attention hyperactivity"). * Details are described in Supplementary Table S1; ** Current study samples

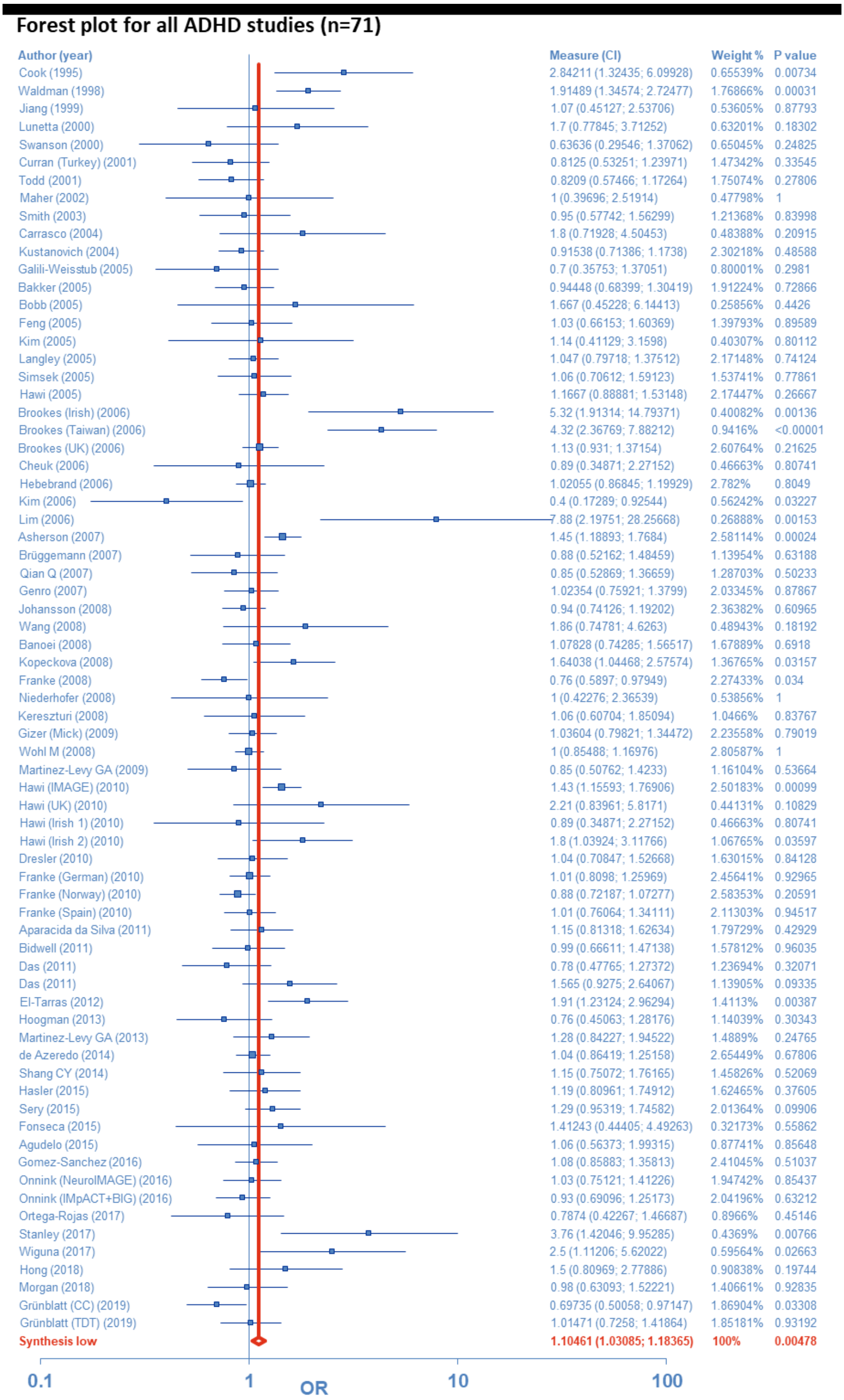

Supplementary Figure S2 Meta-analysis of all cohorts and published association analyses (n=71) of the *DAT1* 3'-UTR VNTR Long-allele with attention-deficit hyperactivity disorder (ADHD) including ID number, individual OR and 95% CI, individual weight and individual P-value. Black whiskers in the forest plot represent 95% confidence intervals (CI) for odds ratio; the weight (inverse variance) of the study is reflected in symbol (box) size. Sample demographics, individual statistics, heterogeneity, literature bias statistics, quality assessments and scores, and model used is summarized in supplementary tables S3, S5-S6.

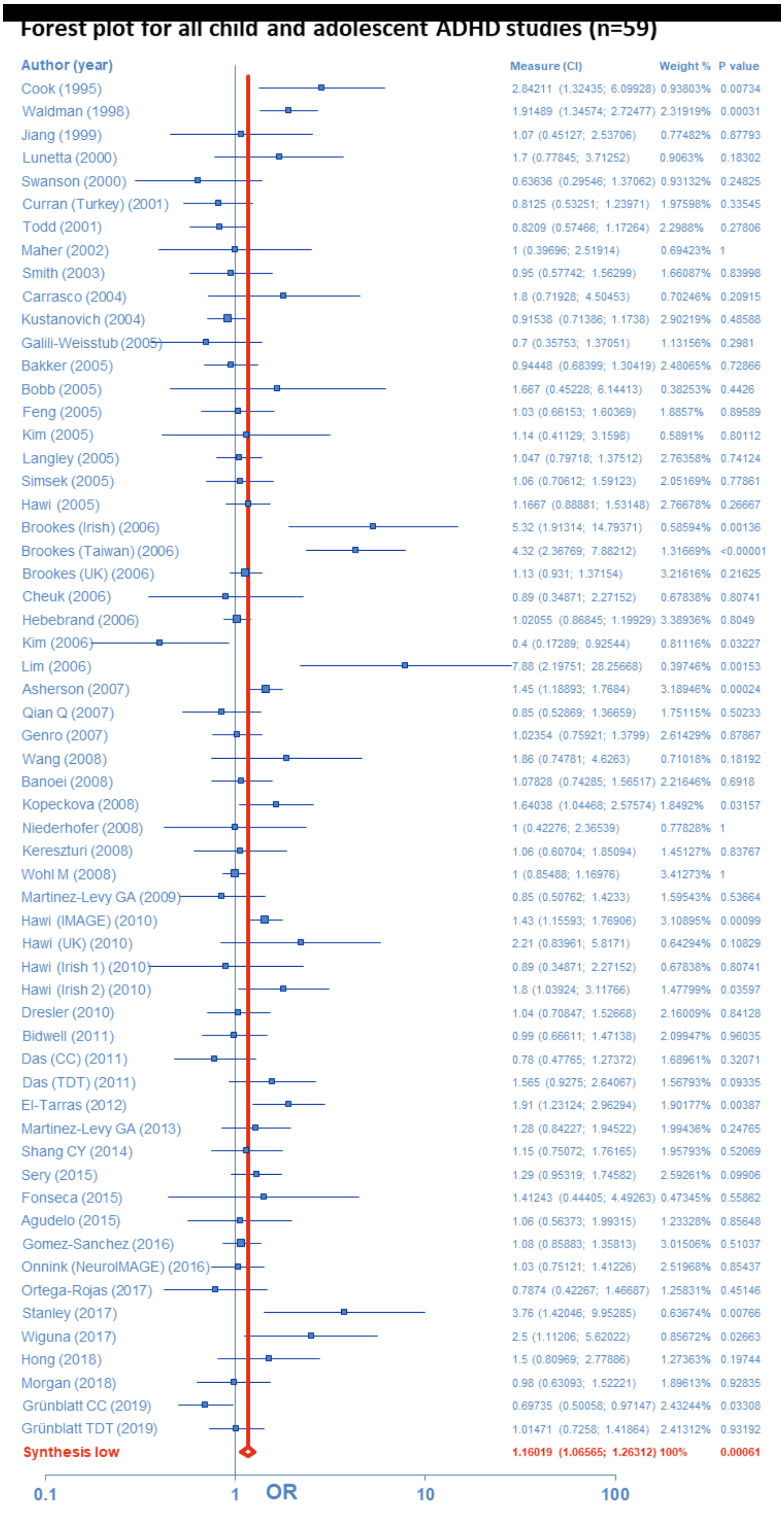

Supplementary Figure S3 Meta-analysis in child and adolescent ADHD studies (n=59) of the *DAT1* 3'-UTR VNTR Long-allele with attention-deficit hyperactivity disorder (ADHD) including ID number, individual OR and 95% CI, individual weight and individual P-value. Black whiskers in the forest plot represent 95% confidence intervals (CI) for odds ratio; the weight (inverse variance) of the study is reflected in symbol (box) size. Sample demographics, individual statistics, heterogeneity, literature bias statistics, quality assessments and scores, and model used is summarized in supplementary tables S3, S5-S6.

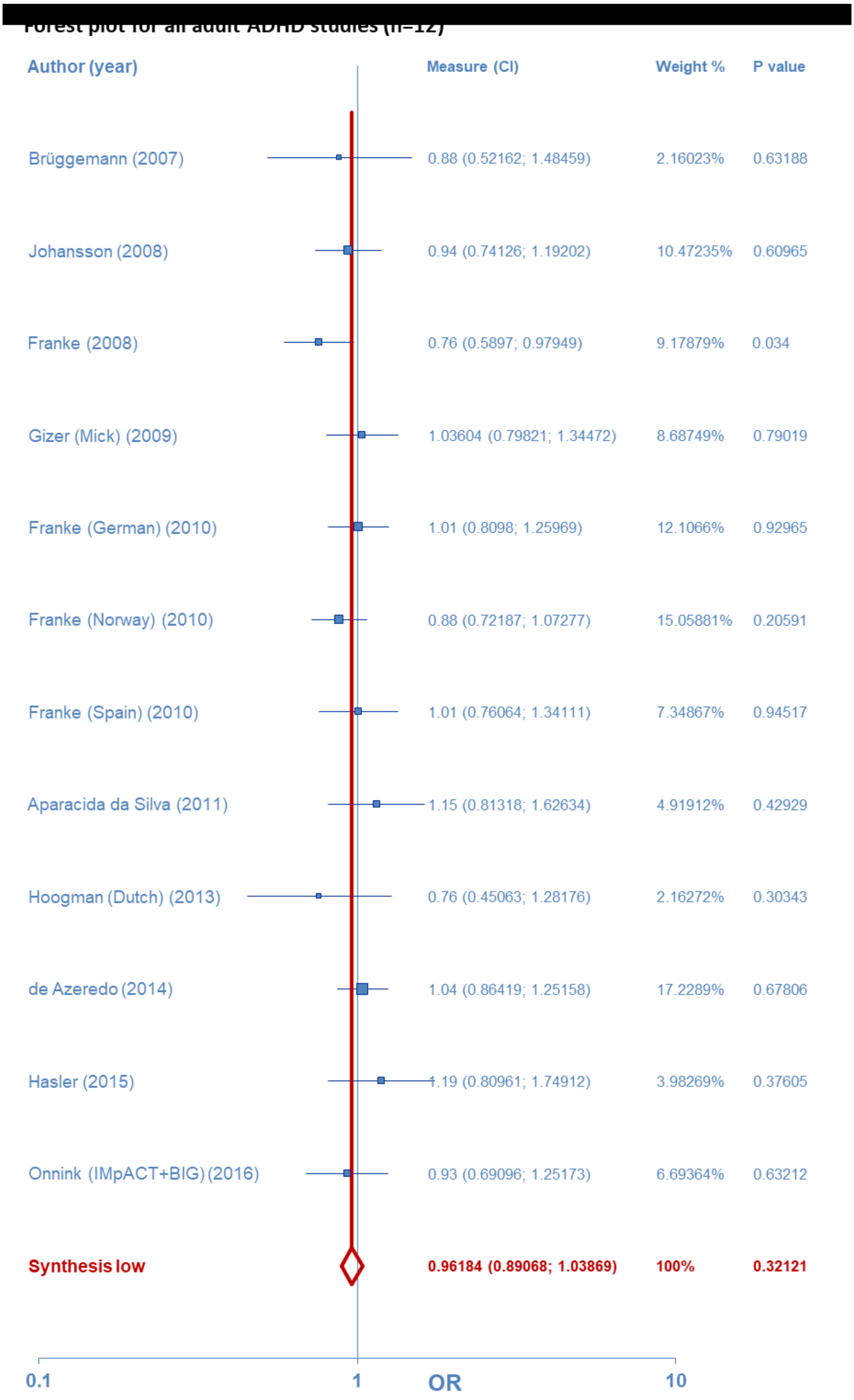

Supplementary Figure S4 Meta-analysis in adult ADHD studies (n=12) of the *DAT1* 3'-UTR VNTR Long-allele with attention-deficit hyperactivity disorder (ADHD) including ID number, individual OR and 95% CI, individual weight and individual P-value. Black whiskers in the forest plot represent 95% confidence intervals (CI) for odds ratio; the weight (inverse variance) of the study is reflected in symbol (box) size. Sample demographics, individual statistics, heterogeneity, literature bias statistics, quality assessments and scores, and model used is summarized in supplementary tables S3, S5-S6.

References

Adriani W, Romano E, Pucci M, Pascale E, Cerniglia L, Cimino S, Tambelli R, Curatolo P, Granstrem O, Maccarrone M, Laviola G, D'Addario C (2018) Potential for diagnosis versus therapy monitoring of attention deficit hyperactivity disorder: a new epigenetic biomarker interacting with both genotype and auto-immunity. Eur Child Adolesc Psych 27 (2):241-252. doi:10.1007/s00787-017-1040-9

Agudelo JA, Galvez JM, Fonseca DJ, Mateus HE, Talero-Gutierrez C, Velez-Van-Meerbeke A (2015) Evidence of an association between 10/10 genotype of DAT1 and endophenotypes of attention deficit/hyperactivity disorder. Neurologia 30 (3):137-143

Aparecida da Silva M, Cordeiro Q, Louza M, Vallada H (2011) Lack of association between a 3'UTR VNTR polymorphism of dopamine transporter gene (SLC6A3) and ADHD in a Brazilian sample of adult patients. J Atten Disord 15 (4):305-309. doi:10.1177/1087054710365989

Asherson P, Brookes K, Franke B, Chen W, Gill M, Ebstein RP, Buitelaar J, Banaschewski T, Sonuga-Barke E, Eisenberg J, Manor I, Miranda A, Oades RD, Roeyers H, Rothenberger A, Sergeant J, Steinhausen HC, Faraone SV (2007) Confirmation that a specific haplotype of the dopamine transporter gene is associated with combined-type ADHD. The American journal of psychiatry 164 (4):674-677. doi:10.1176/ajp.2007.164.4.674

Bakker SC, van der Meulen EM, Oteman N, Schelleman H, Pearson PL, Buitelaar JK, Sinke RJ (2005) DAT1, DRD4, and DRD5 polymorphisms are not associated with ADHD in Dutch families. Am J Med Genet B 132B (1):50-52. doi:10.1002/ajmg.b.30089

Banoei MM, Majidizadeh T, Shirazi E, Moghimi N, Ghadiri M, Najmabadi H, Ohadi M (2008) No Association Between the DAT1 10-Repeat Allele and ADHD in the Iranian Population. Am J Med Genet B 147B (1):110-111. doi:10.1002/ajmg.b.30578

Barr CL, Xu C, Kroft J, Feng Y, Wigg K, Zai G, Tannock R, Schachar R, Malone M, Roberts W, Nothen MM, Grunhage F, Vandenbergh DJ, Uhl G, Sunohara G, King N, Kennedy JL (2001) Haplotype study of three polymorphisms at the dopamine transporter locus confirm linkage to attention-deficit/hyperactivity disorder. Biol Psychiatry 49 (4):333-339. doi:10.1016/s0006-3223(00)01053-2

Bidwell LC, Willcutt EG, McQueen MB, DeFries JC, Olson RK, Smith SD, Pennington BF (2011) A Family Based Association Study of DRD4, DAT1, and 5HTT and Continuous Traits of Attention-Deficit Hyperactivity Disorder. Behav Genet 41 (1):165-174. doi:10.1007/s10519-010-9437-y

Bobb AJ, Addington AM, Sidransky E, Gornick MC, Lerch JP, Greenstein DK, Clasen LS, Sharp WS, Inoff-Germain G, Wavrant-De Vrieze F, Arcos-Burgos M, Straub RE, Hardy JA, Castellanos FX, Rapoport JL (2005) Support for association between ADHD and two candidate genes: NET1 and DRD1. American journal of medical genetics Part B, Neuropsychiatric genetics : the official publication of the International Society of Psychiatric Genetics 134b (1):67-72. doi:10.1002/ajmg.b.30142

Bonvicini C, Faraone SV, Scassellati C (2016) Attention-deficit hyperactivity disorder in adults: A systematic review and meta-analysis of genetic, pharmacogenetic and biochemical studies. Mol Psychiatr 21 (7):872-884. doi:10.1038/mp.2016.74

Brookes K, Xu X, Chen W, Zhou K, Neale B, Lowe N, Anney R, Franke B, Gill M, Ebstein R, Buitelaar J, Sham P, Campbell D, Knight J, Andreou P, Altink M, Arnold R, Boer F, Buschgens C, Butler L, Christiansen H, Feldman L, Fleischman K, Fliers E, Howe-Forbes R, Goldfarb A, Heise A, Gabriels I, Korn-Lubetzki I, Johansson L, Marco R, Medad S, Minderaa R, Mulas F, Muller U, Mulligan A, Rabin K, Rommelse N, Sethna V, Sorohan J, Uebel H, Psychogiou L, Weeks A, Barrett R, Craig I, Banaschewski T, Sonuga-Barke E, Eisenberg J, Kuntsi J, Manor I, McGuffin P, Miranda A, Oades RD, Plomin R, Roeyers H, Rothenberger A, Sergeant J, Steinhausen HC, Taylor E, Thompson M, Faraone SV, Asherson P (2006) The analysis of 51 genes in DSM-IV combined type attention deficit hyperactivity disorder: association signals in DRD4, DAT1 and 16 other genes. Mol Psychiatry 11 (10):934-953. doi:10.1038/sj.mp.4001869

Brown AB, Biederman J, Valera E, Makris N, Doyle A, Whitfield-Gabrieli S, Mick E, Spencer T, Faraone S, Seidman L (2011) Relationship of DAT1 and adult ADHD to task-positive and task-negative working memory networks. Psychiatry Res 193 (1):7-16. doi:10.1016/j.pscychresns.2011.01.006

Brüggemann D, Sobanski E, Alm B, Schubert T, Schmalzried H, Philipsen A, Breen G, Becker T, Georgi A, Skowronek MH, Schulze TG, Treutlein J, Rietschel M (2007) No association between a common haplotype of the 6 and 10-repeat alleles in intron 8 and the 3'UTR of the DAT1 gene and adult attention deficit hyperactivity disorder. Psychiatr Genet 17 (2):121. doi:10.1097/YPG.0b013e32801231d4

Carrasco X, Rothhammer P, Moraga M, Henriquez H, Aboitiz F, Rothhammer F (2004) Presence of DRD4/7R and DAT1/10R allele in Chilean family members with attention deficit hyperactivity disorder. Rev Medica Chile 132 (9):1047-1052

Carrasco X, Rothhammer P, Moraga M, Henriquez H, Chakraborty R, Aboitiz F, Rothhammer F (2006) Genotypic interaction between DRD4 and DAT1 loci is a high risk factor for attention-deficit/hyperactivity disorder in Chilean families. Am J Med Genet B 141B (1):51-54. doi:10.1002/ajmg.b.30259

Chen CK, Chen SL, Mill J, Huang YS, Lin SK, Curran S, Purcell S, Sham P, Asherson P (2003) The dopamine transporter gene is associated with attention deficit hyperactivity disorder in a Taiwanese sample. Mol Psychiatr 8 (4):393-396. doi:10.1038/sj.mp.4001238

Cheuk DK, Li SY, Wong V (2006) No association between VNTR polymorphisms of dopamine transporter gene and attention deficit hyperactivity disorder in Chinese children. American journal of medical genetics Part B, Neuropsychiatric genetics : the official publication of the International Society of Psychiatric Genetics 141b (2):123-125. doi:10.1002/ajmg.b.30280

Cook EH, Stein MA, Krasowski MD, Cox NJ, Olkon DM, Kieffer JE, Leventhal BL (1995) ASSOCIATION OF ATTENTION-DEFICIT DISORDER AND THE DOPAMINE TRANSPORTER GENE. Am J Hum Genet 56 (4):993-998

Cornish KM, Manly T, Savage R, Swanson J, Morisano D, Butler N, Grant C, Cross G, Bentley L, Hollis CP (2005) Association of the dopamine transporter (DAT1) 10/10-repeat genotype with ADHD symptoms and response inhibition in a general population sample. Mol Psychiatr 10 (7):686-698. doi:10.1038/sj.mp.4001641

Costa A, Riedel M, Muller U, Moller HJ, Ettinger U (2011) Relationship between SLC6A3 genotype and striatal dopamine transporter availability: a meta-analysis of human single photon emission computed tomography studies. Synapse 65 (10):998-1005. doi:10.1002/syn.20927

Curran S, Mill J, Tahir E, Kent L, Richards S, Gould A, Huckett L, Sharp J, Batten C, Fernando S, Ozbay F, Yazgan Y, Simonoff E, Thompson M, Taylor E, Asherson P (2001) Association study of a dopamine transporter polymorphism and attention deficit hyperactivity disorder in UK and Turkish samples. Mol Psychiatr 6 (4):425-428. doi:10.1038/sj.mp.4000914

Daly G, Hawi Z, Fitzgerald M, Gill M (1999) Mapping susceptibility loci in attention deficit hyperactivity disorder: preferential transmission of parental alleles at DAT1, DBH and DRD5 to affected children. Mol Psychiatr 4 (2):192-196. doi:10.1038/sj.mp.4000510

Das M, Das Bhowmik A, Bhaduri N, Sarkar K, Ghosh P, Sinha S, Ray A, Chatterjee A, Mukhopadhyay K (2011) Role of gene-gene/gene-environment interaction in the etiology of eastern Indian ADHD probands. Prog Neuro-Psychopharmacol Biol Psychiatry 35 (2):577-587. doi:10.1016/j.pnpbp.2010.12.027

Das M, Mukhopadhyay K (2007) DAT1 3 '-UTR 9R Allele: Preferential transmission in Indian children with attention deficit hyperactivity disorder. Am J Med Genet B 144B (6):826-829. doi:10.1002/ajmg.b.30513

de Azeredo LA, Rovaris DL, Mota NR, Polina ER, Marques FZ, Contini V, Vitola ES, Belmonte-de-Abreu P, Rohde LA, Grevet EH, Bau CH (2014) Further evidence for the association between a polymorphism in the promoter region of SLC6A3/DAT1 and ADHD: findings from a sample of adults. Eur Arch Psychiatry Clin Neurosci 264 (5):401-408. doi:10.1007/s00406-014-0486-8

DiMaio S, Grizenko N, Joober R (2003) Dopamine genes and attention-deficit hyperactivity disorder: a review. J Psychiatry Neurosci 28 (1):27-38

Dresler T, Ehlis AC, Heinzel S, Renner TJ, Reif A, Baehne CG, Heine M, Boreatti-Hummer A, Jacob CP, Lesch KP, Fallgatter AJ (2010) Dopamine Transporter (SLC6A3) Genotype Impacts Neurophysiological Correlates of Cognitive Response Control in an Adult Sample of Patients with ADHD. Neuropsychopharmacology 35 (11):2193-2202. doi:10.1038/npp.2010.91

El-Tarras AE, Alsulaimani AA, Awad NS, Mitwaly N, Said MM, Sabry AM (2012) Association study between the dopamine-related candidate gene polymorphisms and ADHD among Saudi Arabia population via PCR technique. Mol Biol Rep 39 (12):11081-11086. doi:10.1007/s11033-012-2012-2

Ettinger U, Merten N, Kambeitz J (2016) Meta-analysis of the association of the SLC6A3 3 '-UTR VNTR with cognition. Neurosci Biobehav Rev 60:72-81. doi:10.1016/j.neubiorev.2015.09.021

Faraone SV, Spencer TJ, Madras BK, Zhang-James Y, Biederman J (2014) Functional effects of dopamine transporter gene genotypes on in vivo dopamine transporter functioning: a meta-analysis. Mol Psychiatr 19 (8):880-889. doi:10.1038/mp.2013.126

Feng Y, Wigg KG, Makkar R, Ickowicz A, Pathare T, Tannock R, Roberts W, Malone M, Kennedy JL, Schachar R, Barr CL (2005) Sequence variation in the 3 '-untranslated region of the dopamine transporter gene and attention-deficit hyperactivity disorder (ADHD). Am J Med Genet B 139B (1):1-6. doi:10.1002/ajmg.b30190

Fonseca DJ, Mateus HE, Galvez JM, Forero DA, Talero-Gutierrez C, Velez-van-Meerbeke A (2015) Lack of association of polymorphisms in six candidate genes in colombian adhd patients. Ann Neurosci 22 (4):217-221. doi:10.5214/ans.0972.7531.220405

Franke B, Hoogman M, Arias Vasquez A, Heister JG, Savelkoul PJ, Naber M, Scheffer H, Kiemeney LA, Kan CC, Kooij JJ, Buitelaar JK (2008) Association of the dopamine transporter (SLC6A3/DAT1) gene 9-6 haplotype with adult ADHD. American journal of medical genetics Part B, Neuropsychiatric genetics : the official publication of the International Society of Psychiatric Genetics 147b (8):1576-1579. doi:10.1002/ajmg.b.30861

Franke B, Vasquez AA, Johansson S, Hoogman M, Romanos J, Boreatti-Hummer A, Heine M, Jacob CP, Lesch KP, Casas M, Ribases M, Bosch R, Sanchez-Mora C, Gomez-Barros N, Fernandez-Castillo N, Bayes M, Halmoy A, Halleland H, Landaas ET, Fasmer OB, Knappskog PM, Heister A, Kiemeney LA, Kooij JJS,

Boonstra AM, Kan CC, Asherson P, Faraone SV, Buitelaar JK, Haavik J, Cormand B, Ramos-Quiroga JA, Reif A (2010) Multicenter Analysis of the SLC6A3/DAT1 VNTR Haplotype in Persistent ADHD Suggests Differential Involvement of the Gene in Childhood and Persistent ADHD. Neuropsychopharmacology 35 (3):656-664. doi:10.1038/npp.2009.170

Galili-Weisstub E, Levy S, Frisch A, Gross-Tsur V, Michaelovsky E, Kosov A, Meltzer A, Goltser T, Serretti A, Cusin C, Darvasi A, Inbar E, Weizman A, Segman RH (2005) Dopamine transporter haplotype and attention-deficit hyperactivity disorder. Mol Psychiatry 10 (7):617-618. doi:10.1038/sj.mp.4001655

Gatt JM, Burton KL, Williams LM, Schofield PR (2015) Specific and common genes implicated across major mental disorders: a review of meta-analysis studies. J Psychiatr Res 60:1-13. doi:10.1016/j.jpsychires.2014.09.014

Genro JP, Polanczyk GV, Zeni C, Oliveira AS, Roman T, Rohde LA, Hutz MH (2008) A Common Haplotype at the Dopamine Transporter Gene 5 ' Region is Associated With Attention-Deficit/Hyperactivity Disorder. Am J Med Genet B 147B (8):1568-1575. doi:10.1002/ajmg.b.30863

Gill M, Daly G, Heron S, Hawi Z, Fitzgerald M (1997) Confirmation of association between attention deficit hyperactivity disorder and a dopamine transporter polymorphism. Mol Psychiatr 2 (4):311-313. doi:10.1038/sj.mp.4000290

Gizer IR, Ficks C, Waldman ID (2009) Candidate gene studies of ADHD: a meta-analytic review. Hum Genet 126 (1):51-90. doi:10.1007/s00439-009-0694-x

Gomez-Sanchez CI, Riveiro-Alvarez R, Soto-Insuga V, Rodrigo M, Tirado-Requero P, Mahillo-Fernandez I, Abad-Santos F, Carballo JJ, Dal-Re R, Ayuso C (2016) Attention deficit hyperactivity disorder: genetic association study in a cohort of Spanish children. Behav Brain Funct 12:10. doi:10.1186/s12993-015-0084-6

Hasler R, Salzmann A, Bolzan T, Zimmermann J, Baud P, Giannakopoulos P, Perroud N (2015) DAT1 and DRD4 genes involved in key dimensions of adult ADHD. Neurol Sci 36 (6):861-869. doi:10.1007/s10072-014-2051-7

Hawi Z, Kent L, Hill M, Anney RJ, Brookes KJ, Barry E, Franke B, Banaschewski T, Buitelaar J, Ebstein R, Miranda A, Oades RD, Roeyers H, Rothenberger A, Sergeant J, Sonuga-Barke E, Steinhausen HC, Faraone SV, Asherson P, Gill M (2010) ADHD and DAT1: further evidence of paternal over-transmission of risk alleles and haplotype. American journal of medical genetics Part B, Neuropsychiatric genetics : the official publication of the International Society of Psychiatric Genetics 153b (1):97-102. doi:10.1002/ajmg.b.30960

Hawi Z, Lowe N, Kirley A, Gruenhage F, Nothen M, Greenwood T, Kelsoe J, Fitzgerald M, Gill M (2003) Linkage disequilibrium mapping at DAT1, DRD5 and DBH narrows the search for ADHD susceptibility alleles at these loci. Mol Psychiatr 8 (3):299-308. doi:10.1038/sj.mp.4001290

Hawi Z, Segurado R, Conroy J, Sheehan K, Lowe N, Kirley A, Shields D, Fitzgerald M, Gallagher L, Gill M (2005) Preferential transmission of paternal alleles at risk genes in attention-deficit/hyperactivity disorder. Am J Hum Genet 77 (6):958-965. doi:10.1086/498174

Hebebrand J, Dempfle A, Saar K, Thiele H, Herpertz-Dahlmann B, Linder M, Kiefl H, Remschmidt H, Hemminger U, Warnke A, Knolker U, Heiser P, Friedel S, Hinney A, Schafer H, Nurnberg P, Konrad K (2006) A genome-wide scan for attention-deficit/hyperactivity disorder in 155 German sib-pairs. Mol Psychiatr 11 (2):196-205. doi:10.1038/sj.mp.4001761

Holmes J, Payton A, Barrett JH, Hever T, Fitzpatrick H, Trumper AL, Harrington R, McGuffin P, Owen M, Ollier W, Worthington J, Thapar A (2000) A family-based and case-control association study of the dopamine D4 receptor gene and dopamine transporter gene in attention deficit hyperactivity disorder. Mol Psychiatr 5 (5):523-530. doi:10.1038/sj.mp.4000751

Hong JH, Hwang IW, Lim MH, Kwon HJ, Jin HJ (2018) Genetic associations between ADHD and dopaminergic genes (DAT1 and DRD4) VNTRs in Korean children. Genes Genom 40 (12):1309-1317. doi:10.1007/s13258-018-0726-9

Hoogman M, Onnink M, Cools R, Aarts E, Kan C, Arias Vasquez A, Buitelaar J, Franke B (2013) The dopamine transporter haplotype and reward-related striatal responses in adult ADHD. European neuropsychopharmacology : the journal of the European College of Neuropsychopharmacology 23 (6):469-478. doi:10.1016/j.euroneuro.2012.05.011

Jiang S, Xin R, Qian Y, Lin S, Tang G, Wang D, Chen M, Ren D (1999) The relationship between attention-deficit hyperactivity disorder and dopamine transporter 1 gene. Chinese Journal Nervous and Mental Disease 25:355-357

Johansson S, Halleland H, Halmoy A, Jacobsen KK, Landaas ET, Dramsdahl M, Fasmer OB, Bergsholm P, Lundervold AJ, Gillberg C, Hugdahl K, Knappskog PM, Haavik J (2008) Genetic Analyses of Dopamine Related Genes in Adult ADHD Patients Suggest an Association With the DRD5-Microsatellite Repeat, But Not With DRD4 or SLC6A3 VNTRs. Am J Med Genet B 147B (8):1470-1475. doi:10.1002/ajmg.b.30662

Kambeitz J, Romanos M, Ettinger U (2014) Meta-analysis of the association between dopamine transporter genotype and response to methylphenidate treatment in ADHD. Pharmacogenomics J 14 (1):77-84. doi:10.1038/tpj.2013.9

Kereszturi E, Tarnok Z, Bognar E, Lakatos K, Farkas L, Gadoros J, Sasvari-Szekely M, Nemoda Z (2008) Catechol-O-Methyltransferase Val158Met Polymorphism Is Associated With Methylphenidate Response in ADHD Children. Am J Med Genet B 147B (8):1431-1435. doi:10.1002/ajmg.b.30704

Kim JW, Kim BN, Cho SC (2006) The dopamine transporter gene and the impulsivity phenotype in attention deficit hyperactivity disorder: A case-control association study in a Korean sample. J Psychiatr Res 40 (8):730-737. doi:10.1016/j.jpsychires.2005.11.002

Kim YS, Leventhal BL, Kim SJ, Kim BN, Cheon KA, Yoo HJ, Kim SJ, Badner J, Cook EH (2005) Family-based association study of DAT1 and DRD4 polymorphism in Korean children with ADHD. Neurosci Lett 390 (3):176-181. doi:10.1016/j.neulet.2005.08.025

Kirley A, Hawi Z, Daly G, McCarron M, Mullins C, Millar N, Waldman I, Fitzgerald M, Gill M (2002) Dopaminergic system genes in ADHD: Toward a biological hypothesis. Neuropsychopharmacology 27 (4):607-619

Kopeckova M, Paclt I, Petrasek J, Pacltova D, Malikova M, Zagatova V (2008) Some ADHD polymorphisms (in genes DAT1, DRD2, DRD3, DBH, 5-HTT) in case-control study of 100 subjects 6-10 age. Neuroendocrinol Lett 29 (2):246-251

Kustanovich V, Ishii J, Crawford L, Yang M, McGough JJ, McCracken JT, Smalley SL, Nelson SF (2004) Transmission disequilibrium testing of dopamine-related candidate gene polymorphisms in ADHD: confirmation of association of ADHD with DRD4 and DRD5. Mol Psychiatr 9 (7):711-717. doi:10.1038/sj.mp.4001466

Langley K, Fowler TA, Grady DL, Moyzis RK, Holmans PA, van den Bree MB, Owen MJ, O'Donovan MC, Thapar A (2009) Molecular genetic contribution to the developmental course of attention-deficit hyperactivity disorder. Eur Child Adolesc Psychiatry 18 (1):26-32. doi:10.1007/s00787-008-0698-4

Langley K, Turic D, Peirce TR, Mills S, Van den Bree MB, Owen MJ, O'Donovan MC, Thapar A (2005) No support for association between the dopamine transporter (DAT1) gene and ADHD. Am J Med Genet B 139B (1):7-10. doi:10.1002/ajmg.b.30206

Li D, Sham PC, Owen MJ, He L (2006) Meta-analysis shows significant association between dopamine system genes and attention deficit hyperactivity disorder (ADHD). Hum Mol Genet 15 (14):2276-2284. doi:10.1093/hmg/ddl152

Lim MH, Kim HW, Paik KC, Cho SC, Yoon DY, Lee HJ (2006) Association of the DAT1 polymorphism with attention deficit hyperactivity disorder (ADHD): A family-based approach. Am J Med Genet B 141B (3):309-311. doi:10.1002/ajmg.b.30282

Liu J, Yang A, Zhang Q, Yang G, Yang W, Lei H, Quan J, Qu F, Wang M, Zhang Z, Yu K (2015) Association between genetic variants in SLC25A12 and risk of autism spectrum disorders: An integrated meta-analysis. Am J Med Genet B Neuropsychiatr Genet 168B (4):236-246. doi:10.1002/ajmg.b.32304

Lunetta KL, Faraone SV, Biederman J, Laird NM (2000) Family-based tests of association and linkage that use unaffected sibs, covariates, and interactions. Am J Hum Genet 66 (2):605-614. doi:10.1086/302782

Maher BS, Marazita ML, Ferrell RE, Vanyukov MM (2002) Dopamine system genes and attention deficit hyperactivity disorder: a meta-analysis. Psychiatr Genet 12 (4):207-215. doi:10.1097/00041444-200212000-00003

Martinez-Levy G, Diaz-Galvis J, Briones-Velasco M, Gomez-Sanchez A, De la Pena-Olvera F, Sosa-Mora L, Palacios-Cruz L, Ricardo-Garcell J, Reyes-Zamorano E, Cruz-Fuentes C (2009) Genetic interaction analysis for DRD4 and DAT1 genes in a group of Mexican ADHD patients. Neurosci Lett 451 (3):257-260. doi:10.1016/j.neulet.2009.01.004

Martinez-Levy GA, Benjet C, Briones-Velasco M, Perez-Molina A, Nani A, Cruz-Fuentes CS (2013) Genetic variability of DRD4 and DAT1 in the urban population of Mexico City. Salud Ment 36 (3):189-192. doi:10.17711/sm.0185-3325.2013.024

Morgan JE, Caplan B, Tung I, Norona AN, Baker BL, Lee SS (2018) COMT and DAT1 polymorphisms moderate the indirect effect of parenting behavior on youth ADHD symptoms through neurocognitive functioning. Child Neuropsychol 24 (6):823-843. doi:10.1080/09297049.2017.1346067

Niederhofer H, Menzel F, Gobel K, Hackenberg B, Richter R, Walter MH, Gross C, Huber M, Pycha R, Menzel HJ (2008) A preliminary report of the dopamine receptor D(4) and the dopamine transporter 1 gene polymorphism and its association with attention deficit hyperactivity disorder. Neuropsychiatr Dis Treat 4 (4):701-705

Nyman ES, Ogdie MN, Loukola A, Varilo T, Taanila A, Hurtig T, Moilanen IK, Loo SK, McGough JJ, Jarvelin MR, Smalley SL, Nelson SF, Peltonen L (2007) ADHD candidate gene study in a population-based birth cohort: association with DBH and DRD2. J Am Acad Child Adolesc Psychiatry 46 (12):1614-1621. doi:10.1097/chi.0b013e3181579682

Onnink AM, Franke B, van Hulzen K, Zwiers MP, Mostert JC, Schene AH, Heslenfeld DJ, Oosterlaan J, Hoekstra PJ, Hartman CA, Vasquez AA, Kan CC, Buitelaar J, Hoogman M (2016) Enlarged striatal volume in adults with ADHD carrying the 9-6 haplotype of the dopamine transporter gene DAT1. Journal of neural transmission (Vienna, Austria : 1996) 123 (8):905-915. doi:10.1007/s00702-016-1521-x

Ortega-Rojas J, Arboleda-Bustos CE, Morales L, Benitez BA, Beltran D, Izquierdo A, Arboleda H, Vasquez R (2017) [Study of genetic variants in the BDNF, COMT, DAT1 and SERT genes in Colombian children with attention deficit disorder]. Revista colombiana de psiquiatria 46 (4):222-228. doi:10.1016/j.rcp.2016.08.006

Palmer CG, Bailey JN, Ramsey C, Cantwell D, Sinsheimer JS, Del'Homme M, McGough J, Woodward JA, Asarnow R, Asarnow J, Nelson S, Smalley SL (1999) No evidence of linkage or linkage disequilibrium between DAT1 and attention deficit hyperactivity disorder in a large sample. Psychiatr Genet 9 (3):157-160

Purper-Ouakil D, Wohl M, Mouren MC, Verpillat R, Ades J, Gorwood R (2005) Meta-analysis of family-based association studies between the dopamine transporter gene and attention deficit hyperactivity disorder. Psychiatr Genet 15 (1):53-59. doi:10.1097/00041444-200503000-00009

Qian Q, Wang Y, Li J, Yang L, Wang B, Zhou R, Glatt SJ, Faraone SV (2007) Evaluation of potential gene-gene interactions for attention deficit hyperactivity disorder in the Han Chinese population. American journal of medical genetics Part B, Neuropsychiatric genetics : the official publication of the International Society of Psychiatric Genetics 144b (2):200-206. doi:10.1002/ajmg.b.30422

Qian Q, Wang Y, Zhou R, Yang L, Faraone SV (2004) Family-based and case-control association studies of DRD4 and DAT1 polymorphisms in Chinese attention deficit hyperactivity disorder patients suggest long repeats contribute to genetic risk for the disorder. American journal of medical genetics Part B, Neuropsychiatric genetics : the official publication of the International Society of Psychiatric Genetics 128b (1):84-89. doi:10.1002/ajmg.b.30079

Roman T, Schmitz M, Polanczyk G, Eizirik M, Rohde LA, Hutz MH (2001) Attention-deficit hyperactivity disorder: A study of association with both the dopamine transporter gene and the dopamine D4 receptor gene. Am J Med Genet 105 (5):471-478. doi:10.1002/ajmg.1408

Rowe DC, Stever C, Chase D, Sherman S, Abramowitz A, Waldman ID (2001) Two dopamine genes related to reports of childhood retrospective inattention and conduct disorder symptoms. Mol Psychiatr 6 (4):429-433. doi:10.1038/sj.mp.4000874

Sery O, Paclt I, Drtilkova I, Theiner P, Kopeckova M, Zvolsky P, Balcar VJ (2015) A 40-bp VNTR polymorphism in the 3 '-untranslated region of DAT1/SLC6A3 is associated with ADHD but not with alcoholism. Behav Brain Funct 11:8. doi:10.1186/s12993-015-0066-8

Shang CY, Gau SS (2014) Association between the DAT1 gene and spatial working memory in attention deficit hyperactivity disorder. The international journal of neuropsychopharmacology 17 (1):9-21. doi:10.1017/s1461145713000783

Shang CY, Gau SS, Liu CM, Hwu HG (2011) Association between the dopamine transporter gene and the inattentive subtype of attention deficit hyperactivity disorder in Taiwan. Prog Neuropsychopharmacol Biol Psychiatry 35 (2):421-428. doi:10.1016/j.pnpbp.2010.08.016

Simsek M, Al-Sharbati M, Al-Adawi S, Ganguly SS, Lawatia K (2005) Association of the risk allele of dopamine transporter gene (DAT1*10) in Omani male children with attention-deficit hyperactivity disorder. Clin Biochem 38 (8):739-742. doi:10.1016/j.clinbiochem.2005.04.016

Smith KM, Daly M, Fischer M, Yiannoutsos CT, Bauer L, Barkley R, Navia BA (2003) Association of the dopamine beta hydroxylase gene with attention deficit hyperactivity disorder: Genetic analysis of the Milwaukee longitudinal study. Am J Med Genet B 119B (1):77-85. doi:10.1002/ajmg.b.20005

Soleimani R, Salehi Z, Soltanipour S, Hasandokht T, Jalali MM (2018) SLC6A3 polymorphism and response to methylphenidate in children with ADHD: A systematic review and meta-analysis. Am J Med Genet B 177 (3):287-300. doi:10.1002/ajmg.b.32613

Spencer TJ, Biederman J, Faraone SV, Madras BK, Bonab AA, Dougherty DD, Batchelder H, Clarke A, Fischman AJ (2013) Functional Genomics of Attention-Deficit/Hyperactivity Disorder (ADHD) Risk Alleles on Dopamine Transporter Binding in ADHD and Healthy Control Subjects. Biol Psychiatry 74 (2):84-89. doi:10.1016/j.biopsych.2012.11.010

Stanley A, Chavda K, Subramanian A, Prabhu SV, Ashavaid TF (2017) DRD4 and DAT1 VNTR Genotyping in Children with Attention Deficit Hyperactivity Disorder. Indian J Clin Biochem 32 (2):239-242. doi:10.1007/s12291-016-0587-4

Swanson JM, Flodman P, Kennedy S, Spence MA, Moyzis R, Schuck S, Murias M, Moriarity J, Barr C, Smith M, Posner M (2000) Dopamine genes and ADHD. Neurosci Biobehav Rev 24 (1):21-25. doi:10.1016/s0149-7634(99)00062-7

Todd RD, Huang H, Smalley SL, Nelson SF, Willcutt EG, Pennington BF, Smith SD, Faraone SV, Neuman RJ (2005) Collaborative analysis of DRD4 and DAT genotypes in population-defined ADHD subtypes. Journal of child psychology and psychiatry, and allied disciplines 46 (10):1067-1073. doi:10.1111/j.1469-7610.2005.01517.x

Todd RD, Jong YJ, Lobos EA, Reich W, Heath AC, Neuman RJ (2001) No association of the dopamine transporter gene 3' VNTR polymorphism with ADHD subtypes in a population sample of twins. Am J Med Genet 105 (8):745-748

van Rooij D, Hoekstra PJ, Bralten J, Hakobjan M, Oosterlaan J, Franke B, Rommelse N, Buitelaar JK, Hartman CA (2015) Influence of DAT1 and COMT variants on neural activation during response inhibition in

adolescents with attention-deficit/hyperactivity disorder and healthy controls. Psychol Med 45 (15):3159-3170. doi:10.1017/s0033291715001130

Waldman ID, Rowe DC, Abramowitz A, Kozel ST, Mohr JH, Sherman SL, Cleveland HH, Sanders ML, Card JHC, Stever C (1998) Association and linkage of the dopamine transporter gene and attention-deficit hyperactivity disorder in children: Heterogeneity owing to diagnostic subtype and severity. Am J Hum Genet 63 (6):1767-1776. doi:10.1086/302132

Wang Y, Wang Z, Yao K, Tanaka K, Yang Y, Shirakawa O, Maeda K (2008) Lack of association between the dopamine transporter gene 3' VNTR polymorphism and attention deficit hyperactivity disorder in Chinese Han children: case-control and family-based studies. The Kobe journal of medical sciences 53 (6):327-333

Wang Z, Wang Y, Yao K, Yang Y, Liu L (2004) Survey the association between dopamine transporter gene polymorphism and attention def icit hyperactivity disorder. Chinese Journal of child health care 12:289-292

Wiguna T, Ismail RI, Winarsih NS, Kaligis F, Hapsari A, Budiyanti L, Sekartini R, Rahayu S, Guerrero APS (2017) Dopamine transporter gene polymorphism in children with ADHD: A pilot study in Indonesian samples. Asian J Psychiatr 29:35-38. doi:10.1016/j.ajp.2017.03.041

Wohl M, Boni C, Asch M, Cortese S, Orejarena S, Mouren MC, Gorwood P, Purper-Ouakil D (2008) Lack of association of the dopamine transporter gene in a French ADHD sample. American journal of medical genetics Part B, Neuropsychiatric genetics : the official publication of the International Society of Psychiatric Genetics 147b (8):1509-1510. doi:10.1002/ajmg.b.30695

Yang B, Chan RC, Jing J, Li T, Sham P, Chen RY (2007) A meta-analysis of association studies between the 10-repeat allele of a VNTR polymorphism in the 3'-UTR of dopamine transporter gene and attention deficit hyperactivity disorder. American journal of medical genetics Part B, Neuropsychiatric genetics : the official publication of the International Society of Psychiatric Genetics 144b (4):541-550. doi:10.1002/ajmg.b.30453
