# Supplementary material for: Association study and a systematic meta-analysis of the VNTR polymorphism in the 3′-UTR of dopamine transporter gene and attention-deficit hyperactivity disorder
Source: J Neural Transm (Vienna). 2019 Mar 28;126(4):517–29. doi: 10.1007/s00702-019-01998-x (PMC6456487; doi:10.1007/s00702-019-01998-x)
Supplement: Supplementary file 2 — Supplementary material 2 (PDF 438 KB) [file 702_2019_1998_MOESM2_ESM.pdf]

Supplenetary Table S2 Summary statistics for meta-analysis of DAT1 3'-UTR VNTR 10-repeat allele as risk allele versus 9-repeat allele, in all ADHD samples as well as stratified by age or entnicity. Including heterogeneity statistics, literature bias analysis followed by Trimm and Fill corrections.

	Total no. Studies		Heterogeneity statistics (I2)				Synthesis model		Synthesis details						Begg's test						Egger's regression test (intercept)						Trim and fill correction		
**ID included**	**included**	**Defenition**	**Estimate**	**CI-**	**CI+**	**p-value**	**Fixed**	**Random**	**N**	**OR**	**ci-**	**ci+**	**z**	**p**	**tau b**	**k**	**S (P-Q)**	**ties**	**z**	**p**	**coef**	**se**	**ci-**	**ci+**	**z**	**p**	**OR**	**ci-**	**ci+**
1-71	71	all ADHD	50.003%	34.19%	62.017%	0		Yes	40129	1.0552	0.9880	1.1269	1.6001	0.1096	0.1328	71	331	0	1.6380	0.1014	0.3364	0.3378	-0.3258	0.9984	0.9957	0.3194			
28, 31, 35, 38, 46-49, 54, 56, 58, 64	12	Adult ADHD	0%	0%	58.316%	0.53629	Yes		9047	0.9368	0.8673	1.0119	-1.6588	0.0972	-0.2576	12	-18	0	-1.1657	0.2437	-1.1950	0.9114	-2.9812	0.5913	-1.3112	0.1898			
28, 31, 35, 46-49, 54, 56, 58, 64	11	Adult ADHD (Excl. Gizer)	0%	0%	60.229%	0.50324	Yes		8041	0.9278	0.8558	1.0058	-1.8189	0.0689	-0.2909	11	-17	0	-1.2456	0.2129	-1.2117	0.9222	-3.0192	0.5959	-1.3138	0.1889			
28, 31, 35, 46-48, 54, 58, 64	9	Adult ADHD-EU	0%	0%	64.798%	0.61714	Yes		6423	**0.8909**	**0.8118**	**0.9777**	-2.4356	**0.0149**	-0.2500	9	-10	0	-0.9383	0.3481	-1.1460	0.9673	-3.0419	0.7499	-1.1848	0.2361			
49, 56	2	Adult ADHD-Brazil	0%	#NV	#NV	0.81348	Yes		1618	1.0511	0.8930	1.2371	0.5989	0.5492	#NV	#NV	#NV	#NV	#NV	#NV	#NV	#NV	#NV	#NV	#NV	#NV			
1-27, 29-30, 32-34, 36-37, 39-45, 50-53, 55, 57, 59-63, 65-71	59	Children & Adolescent ADHD	51.045%	33.93%	63.726%	**0**		Yes	31082	**1.1050**	**1.0203**	**1.1968**	2.4545	**0.0141**	0.1356	59	233	0	1.5172	0.1292	0.3084	0.3569	-0.3911	1.0078	0.8641	0.3875			
1-2, 4-9, 11-15, 17, 19, 21-22, 24, 27, 34, 36-37, 39, 41-45, 50, 59, 62-63, 69-71	35	Children & Adolescent ADHD-Caucasian	48.545%	23.743%	65.28%	**0.0008**		Yes	23715	**1.1102**	**1.0225**	**1.2055**	2.4885	**0.0128**	0.1546	35	93	0	1.3065	0.1914	0.3597	0.4937	-0.6079	1.3273	0.7286	0.4663			
1-2, 4-5, 7-9, 11, 14-15, 50, 69	12	Children & Adolescent ADHD- North America	55.367%	14.633%	76.664%	**0.01027**		Yes	6037	1.1069	0.8996	1.3620	0.9598	0.3372	0.2273	12	16	0	1.0286	0.3037	0.9838	1.0625	-1.0986	3.0661	0.9259	0.3545			
13, 17, 19, 21-22, 24, 27, 34,36-37, 39, 41-45, 59, 62-63, 70-71	21	Children & Adolescent ADHD- Europe	48.525%	14.587%	68.978%	**0.00695**		Yes	17141	**1.1301**	**1.0316**	**1.2379**	2.6298	**0.0085**	0.1857	21	40	0	1.1777	0.2389	0.5349	0.6690	-0.7763	1.8461	0.7996	0.4240			
6,12, 18, 33, 53	5	Children & Adolescent ADHD- Middle East	0%	0%	79.204%	0.58547	Yes		1081	0.9660	0.7902	1.1808	-0.3381	0.7353	-0.3000	5	-4	0	-0.7349	0.4624	-2.9339	2.0409	-6.9340	1.0663	-1.4375	0.1506			
10, 30, 40, 55, 60-61, 65	7	Children & Adolescent ADHD- South America	0%	0%	70.809%	0.6861	Yes		2252	1.0573	0.8744	1.2785	0.5748	0.5654	0.1905	7	5	0	0.6008	0.5480	0.5248	0.8261	-1.0944	2.1440	0.6352	0.5253			
3, 16, 20, 23, 25-26, 29,32, 51-52, 57, 66-68	14	Children & Adolescent ADHD- Asia	71.666%	51.39%	83.484%	**0.00001**		Yes	4571	1.2233	0.8233	1.8175	0.9977	0.3185	0.2418	14	23	0	1.2044	0.2284	2.0196	1.7415	-1.3937	5.4329	1.1597	0.2462			
3, 16, 20, 23, 25-26, 29,32, 57, 68	10	Children & Adolescent ADHD-Chinese	69.224%	40.734%	84.019%	**0.00059**		Yes	3829	1.1846	0.7620	1.8414	0.7525	0.4517	0.1778	10	9	0	0.7155	0.4743	1.4164	1.8866	-2.2813	5.1141	0.7508	0.4528			
51-52, 66-67	4	Children & Adolescent ADHD- Indo-Asia	81.934%	53.245%	93.02%	**0.00085**		Yes	742	1.3567	0.4990	3.6886	0.5979	0.5499	0.5000	4	4	0	1.0191	0.3082	8.8778	6.6437	-4.1436	21.8991	1.3363	0.1815			
