# Supplementary material for: Association study and a systematic meta-analysis of the VNTR polymorphism in the 3′-UTR of dopamine transporter gene and attention-deficit hyperactivity disorder
Source: J Neural Transm (Vienna). 2019 Mar 28;126(4):517–29. doi: 10.1007/s00702-019-01998-x (PMC6456487; doi:10.1007/s00702-019-01998-x)
Supplement: Supplementary file 3 — Supplementary material 3 (PDF 439 KB) [file 702_2019_1998_MOESM3_ESM.pdf]

Supplenetary Table S2 Summary statistics for meta-analysis of DAT1 3'-UTR VNTR Long-repeat allele as risk allele versus Short-repeat allele, in all ADHD samples as well as stratified by age or entnicity. Including heterogeneity statistics, literature bias analysis followed by Trimm and Fill corrections.

ID included	Total no. Studies included	Defenition	Heterogeneity statistics (I2)				Synthesis model		Synthesis details						Begg's test						Egger's regression test (intercept)						Trim and fill correction		
			Estimate	CI-	CI+	p-value	Fixed	Random	N	OR	ci-	ci+	z	p	tau b	k	S (P-Q)	ties	z	p	coef	se	ci-	ci+	z	p	OR	ci-	ci+
1-71	71	all ADHD	54.167%	40.029%	64.972%	**0**		Yes	40681	**1.1046**	**1.0309**	**1.1837**	2.8217	**0.0048**	0.2016	71	502	4	2.4868	**0.0129**	0.8085	0.3583	0.1062	1.5107	2.2565	0.0240	**1.0614**	**1.0186**	**1.1060**
28, 31, 35, 38, 46-49, 54, 56, 58, 64	12	Adult ADHD	0%	0%	58.316%	0.66306	Yes		9224	0.9618	0.8907	1.0387	-0.9920	0.3212	-0.0152	12	0	0	-0.0686	0.9453	-0.0782	0.9275	-1.8961	1.7397	-0.0843	0.9328			
28, 31, 35, 46-49, 54, 56, 58, 64	11	Adult ADHD (Excl. Gizer)	0%	0%	60.229%	0.60818	Yes		8218	0.9551	0.8813	1.0351	-1.1202	0.2627	-0.0919	0.96	-1.9697	1.79	-0.0959	0.9236	0	11	-1	0	0	1			
28, 31, 35, 46-48, 54, 58, 64	9	Adult ADHD-EU	0%	0%	64.798%	0.67386	Yes		6574	0.9227	0.8413	1.0121	-1.7050	0.0882	-0.0278	9	-2	0	-0.1043	0.9170	0.06691	1.03432	-1.96032	2.09414	0.06469	0.94842			
49, 56	2	Adult ADHD-Brazil	0%	#NV	#NV	0.61602	Yes		1644	1.0635	0.9032	1.2522	0.7386	0.4601	#NV	#NV	#NV	#NV	#NV	#NV	#NV	#NV	#NV	#NV	#NV	#NV			
1-27, 29-30, 32-34, 36-37, 39-45, 50- 53, 55, 57, 59-63, 65-71	59	Children & Adolescent ADHD	56.286%	41.498%	67.335%	**0**		Yes	31457	**1.1602**	**1.0657**	**1.2631**	3.4261	**0.0006**	0.2075	59	356	4	2.3215	**0.0203**	0.7188	0.3901	-0.0458	1.4833	1.8427	0.0654	**1.1053**	**1.0528**	**1.1605**
1-2, 4-9, 11-15, 17, 19, 21-22, 24, 27, 34, 36-37, 39, 41-45, 50, 59, 62-63, 69-71	35	Children & Adolescent ADHD-Caucasian	58.517%	39.736%	71.445%	**0.00001**		Yes	23765	**1.1310**	**1.0282**	**1.2441**	2.5309	**0.0114**	0.1210	35	73	0	1.0225	0.3065	0.46622	0.56504	-0.64123	1.57367	0.82511	0.40931			
1-2, 4-5, 7-9, 11, 14-15, 50, 69	12	Children & Adolescent ADHD- North America	55.107%	14.073%	76.546%	**0.01077**		Yes	6037	1.1146	0.8995	1.3811	0.9919	0.3213	0.2273	12	16	0	1.0286	0.3037	0.95126	1.08017	-1.16584	3.06835	0.88066	0.3785			
13, 17, 19, 21-22, 24, 27, 34,36-37, 39, 41-45, 59, 62-63, 70- 71	21	Children & Adolescent ADHD- Europe	62.116%	39.264%	76.37%	**0.00009**		Yes	17191	**1.1661**	**1.0448**	**1.3015**	2.7417	**0.0061**	0.1381	21	30	0	0.8757	0.3812	0.97244	0.80716	-0.60957	2.55445	1.20477	0.22829			
6,12, 18, 33, 53	5	Children & Adolescent ADHD- Middle East	59.255%	0%	84.794%	**0.04362**		Yes	1249	1.0742	0.7856	1.4689	0.4486	0.6538	-0.1000	5	-2	0	-0.2450	0.8065	-2.36579	4.34956	-10.8908	6.15918	-0.54392	0.5865			
10, 30, 40, 55, 60-61, 65	7	Children & Adolescent ADHD- South America	0%	0%	70.809%	0.6861	Yes		2252	1.0573	0.8744	1.2785	0.5748	0.5654	0.1905	7	5	0	0.6008	0.5480	0.52479	0.82614	-1.09442	2.144	0.63523	0.52528			
3, 16, 20, 23, 25-26, 29,32, 51-52, 57, 66- 68	14	Children & Adolescent ADHD- Asia	66.817%	41.829%	81.071%	**0.00019**		Yes	4728	1.4266	1.0211	1.9932	2.0823	**0.0373**	0.4176	14	39	0	2.0803	**0.0375**	2.57856	1.26416	0.10086	5.05627	2.03975	**0.04138**	1.0991	0.9240	1.3074
3, 16, 20, 23, 25-26, 29,32, 57, 68	10	Children & Adolescent ADHD-Chinese	66.662%	35.004%	82.9%	**0.0014**		Yes	3973	1.3376	0.8819	2.0290	1.3685	0.1712	0.4444	10	21	0	1.7889	0.0736	2.03517	1.51111	-0.92655	4.9969	1.34681	0.17804			
51-52, 66-67	4	Children & Adolescent ADHD- Indo-Asia	74.05%	27.469%	90.715%	**0.00905**		Yes	755	1.6863	0.8809	3.2281	1.5773	0.1147	0.8333	4	6	0	1.6984	0.0894	5.55301	2.19083	1.25906	9.84697	2.53466	**0.01126**	1.0803	0.8184	1.4261
